# Benzoxaborole-modified azithromycins inhibit translation without inducing *ermC* expression

**DOI:** 10.1128/aac.01539-25

**Published:** 2026-03-24

**Authors:** Inna A. Volynkina, Michael O. Bortyazh, Chih-Wei Chen, Andrey G. Tereshchenkov, Anastasiia O. Karakchieva, Dmitrii A. Lukianov, Ekaterina S. Komarova, Alexey E. Tupikin, Dmitry A. Skvortsov, Anna N. Tevyashova, Alexander S. Tikhomirov, Vadim N. Tashlitsky, Marsel R. Kabilov, Andrey E. Shchekotikhin, Olga A. Dontsova, Yury S. Polikanov, Petr V. Sergiev

**Affiliations:** 1 Department of Chemistry, Lomonosov Moscow State University 64935, Moscow, Russia; 2 Center for Molecular and Cellular Biology, Moscow, Russia; 3 A.N. Belozersky Institute of Physico-Chemical Biology, Lomonosov Moscow State University 64935, Moscow, Russia; 4 Department of Biological Sciences, University of Illinois at Chicago 14681, Chicago, Illinois, USA; 5 Institute of Chemical Biology and Fundamental Medicine, Siberian Branch of the Russian Academy of Sciences 104680, Novosibirsk, Novosibirsk Region, Russia; 6 School of Science, Constructor University 84498, Bremen, Germany; 7 Laboratory of Synthesis of Antibiotics Overcoming Drug Resistance, Gause Institute of New Antibiotics 243791, Moscow, Russia; 8 Laboratory of Chemical Transformation of Antibiotics, Gause Institute of New Antibiotics 243791, Moscow, Russia; 9 Department of Functioning of Living Systems, Shemyakin-Ovchinnikov Institute of Bioorganic Chemistry 68485 , Moscow, Russia; 10 Department of Pharmaceutical Sciences, University of Illinois at Chicago 14681, Chicago, Illinois, USA; 11 Center for Biomolecular Sciences, University of Illinois at Chicago 14681, Chicago, Illinois, USA; Columbia University Irving Medical Center, New York, New York, USA

**Keywords:** antibiotic resistance, macrolides, benzoxaboroles, azithromycin derivatives, mechanism of action, translation, context specificity

## Abstract

The rapid increase in antimicrobial resistance underscores the urgent need for new antibacterial agents. One promising strategy involves designing novel compounds through targeted chemical modifications of existing antibiotics. Azithromycin (AZI), a widely used macrolide, has served as a versatile scaffold for developing numerous antibacterial candidates. However, the mechanistic consequences of such modifications remain largely unexplored. Here, we characterize the activity and mechanism of action of three AZI-benzoxaborole (AZI-BB) conjugates. We show that these compounds inhibit bacterial translation *in vitro* and remain active against a model *Escherichia coli* strain carrying an inducible *ermCL-ermC* operon, which confers resistance to macrolide antibiotics. Unlike erythromycin, these derivatives, along with AZI itself, exhibit minimal induction of ErmC expression. Structural analysis reveals that the benzoxaborole moiety of AZI-BB2 forms additional interactions with nucleotides C2441 and C2586 of 23S rRNA, likely contributing to premature ribosome stalling at the *ermCL* regulatory sequence and thereby preventing ErmC expression. Furthermore, high-throughput toeprinting analysis combined with deep sequencing (Toe-seq) demonstrates that AZI-BB2 exhibits reduced sequence specificity for canonical macrolide-sensitive stalling motifs. Altogether, these findings demonstrate that targeted chemical modification of AZI can reshape its context-specific interaction with the ribosome and attenuate the induction of macrolide resistance mechanisms.

## INTRODUCTION

The global spread of antibiotic resistance presents one of the most pressing challenges in modern medicine (1). To address this threat, the development of novel antibacterial agents remains a critical priority. One productive strategy involves chemical modification of clinically validated antibiotics to create derivatives with enhanced potency, broader spectrum of activity, or improved pharmacological and toxicological profiles (2).

Among the available antibacterial drug classes, macrolide antibiotics represent one of the most successful and widely used groups. Azithromycin (AZI), the first semisynthetic 15-membered macrolide, belongs to the azalide subclass and has become a widely used broad-spectrum antibiotic. AZI exhibits activity against both gram-positive and gram-negative bacteria, including clinically relevant pathogens such as *Salmonella enteritidis*, *Neisseria gonorrhoeae*, and various *Staphylococcus* and *Streptococcus* species (3). AZI inhibits bacterial protein synthesis by binding to the nascent peptide exit tunnel (NPET) of the 50S ribosomal subunit, near the peptidyl transferase center (PTC) (4). This interaction leads to context-specific stalling of elongating ribosomes, particularly at sequence motifs encoding (R/K)x(R/K), xPx, and x(R/K)x, where “x” denotes any amino acid residue (5).

The most predominant mechanism of resistance to AZI and other macrolides involves methylation of the universally conserved A2058 nucleotide in the 23S rRNA (*Escherichia coli* numbering), catalyzed by rRNA *N*-methyltransferases of the Erm (erythromycin ribosome methylase) family (6). N6-Dimethylation of A2058 renders the ribosome refractory to macrolide binding, as it disrupts coordination of a water molecule critical for antibiotic accommodation (7). Expression of *erm* genes can be either inducible or constitutive, leading to iMLS_B_ (inducible macrolide-lincosamide-streptogramin B resistant) or cMLS_B_ (constitutive MLS_B_ resistant) phenotypes, respectively (8). Constitutive expression often arises through mutations in the regulatory region of *erm* genes and confers high-level resistance at the cost of reduced translation efficiency and bacterial fitness (9). Therefore, in the absence of sustained antibiotic pressure, bacteria tend to retain inducible *erm* expression, which is regulated by structural rearrangements in *erm* mRNA (8). In the case of *ermC* regulation, the 5′ leader region of its mRNA encodes a short leader peptide (ErmCL) and forms two mutually exclusive stem-loop structures (Fig. S1). Subinhibitory concentrations of macrolide antibiotics promote ribosome stalling at a specific site within the leader open reading frame (ORF), triggering mRNA refolding that exposes the ribosome binding site (RBS) of the downstream *ermC* ORF and activates translation (Fig. S1C).

Owing to its favorable pharmacokinetics and broad-spectrum activity, AZI has frequently been used as a scaffold for the synthesis of third-generation macrolides and hybrid molecules with dual mechanisms of action (2, 10, 11). Chemical modifications at positions C-3, C-5, C-6, N-9a, C-11, C-12, C-2′, C-3′, and C-4″ of the AZI core have produced plenty of derivatives, with some of them showing improved efficacy and resistance-evasion profiles (12–21). For example, 11,12-cyclic carbonate derivatives of AZI display increased activity against *mefA-*expressing erythromycin-resistant strains, which use efflux as a resistance mechanism (18). Similarly, conjugation of aromatic moieties at the C-4″ position has been shown to help circumvent bacterial resistance to macrolides in both *mefA*- and *erm*-expressing strains (18, 22–27).

Macrolones, hybrid molecules linking AZI to fluoroquinolones, have demonstrated potent activity against respiratory pathogens and have proven effective against both inducible and constitutive MLS_B_-type as well as efflux-mediated macrolide resistance (28–39). Macrozones, another class of AZI conjugates featuring thiosemicarbazones, show promise in combating efflux-mediated resistance, although they remain inactive against bacteria with cMLS_B_ phenotype (40).

Unique chemical and physical features of the boron atom gave rise to boron-containing compounds as promising drug candidates for the treatment of a wide range of diseases, including cancer, atopic dermatitis, as well as viral, fungal, and bacterial infections (41–43). Among the diverse boron-containing compounds, benzoxaborole derivatives, developed in part by Anacor Pharmaceuticals Inc., have attracted considerable interest due to their inherent antimicrobial properties, accompanied by low toxicity, chemical stability, and increased reactivity toward *cys*-diol or single hydroxy groups of biological targets (44). With the ready availability of benzoxaboroles on the market, the development of hybrid antibiotics containing this pharmacophore represents a promising direction for circumventing antimicrobial resistance (45).

**Fig 1:**
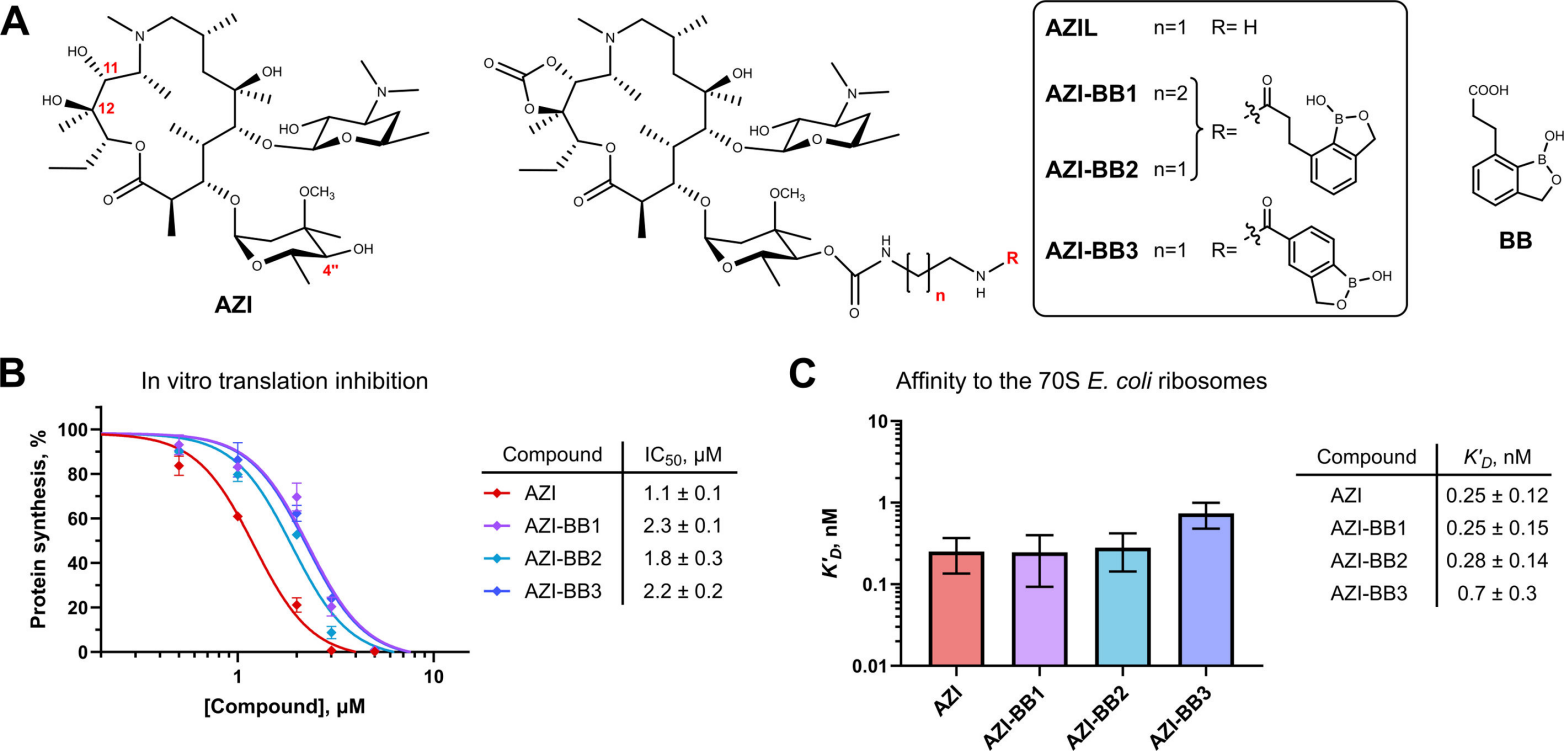
Conjugates of azithromycin with benzoxaboroles bind bacterial ribosome and inhibit translation. (**A**) Chemical structures of compounds used in this work: AZI, azithromycin; AZIL, 4″-*O*-((2-aminoethyl)carbamoyl)−11,12-cyclic carbonate of azithromycin; AZI-BB1-3, conjugates of AZI with benzoxaborole derivatives; BB, benzoxaborole derivative, also known as AN3661 (44). (**B**) AZI-BB conjugates inhibit protein synthesis *in vitro* in a cell-free bacterial translation system. The relative maximum Fluc accumulation rates are shown in the presence of increasing concentrations of AZI and AZI-BB conjugates. Error bars represent standard deviation. The resulting IC_50_ values and 95% confidence intervals are shown in the table. All reactions were repeated at least twice. (**C**) Apparent dissociation constants (*K′_D_*) represent the affinity of the tested compounds for the vacant *E. coli* 70S ribosomes. Error bars represent 95% confidence intervals. The *K′_D_* values and corresponding 95% confidence intervals are shown in the table. For each compound, at least four replicates were performed.

In this study, we investigated a new class of AZI conjugates containing benzoxaborole moieties (AZI-BBs), previously reported to be active against several gram-positive and gram-negative strains (46). From a larger panel of compounds, we selected three lead molecules—AZI-BB1, AZI-BB2, and AZI-BB3 (Fig. 1A)—based on their improved antibacterial profiles. Here, we show that AZI-BB conjugates inhibit bacterial translation by directly interacting with the bacterial ribosome, displaying an affinity comparable to that of AZI. Notably, these compounds also retain activity against a model *E. coli* strain harboring an inducible *ermCL-ermC* operon, which confers resistance to macrolides. Similar to AZI, these compounds are poor inducers of *ermC* expression, with AZI-BB2 and AZI-BB3, in particular, showing a complete lack of induction. Instead of trapping the ribosome at the canonical stall site within the *ermCL* ORF, AZI-BB conjugates cause ribosome arrest upstream of the induction motif, thereby preventing the structural rearrangement required for ErmC production. Using Toe-seq analysis, which represents a combination of toeprinting and next-generation sequencing (NGS) (47), we further demonstrate that AZI-BB2 has a reduced propensity to stall ribosomes at sequence motifs typically sensitive to macrolides. Finally, structural analysis revealed that the benzoxaborole moiety forms additional interactions with the 23S rRNA nucleotides in the NPET, possibly clashing with nascent peptide chains. This previously uncharacterized drug/ribosome contact likely alters the mode of action, underlying the distinct translational arrest profile and resistance-evasion properties of AZI-BB conjugates.

## RESULTS

### AZI-BB conjugates retain activity against inducible *ermC*-expressing *E. coli*

Dozens of AZI derivatives have been synthesized and characterized to date; surprisingly, little is known about how such structural modifications influence the antibiotic’s mechanism of action. In this study, we focused on three promising azithromycin-benzoxaborole conjugates, namely AZI-BB1, AZI-BB2, and AZI-BB3, in which a benzoxaborole moiety is tethered via a linker to the 4″-hydroxy group of AZI’s cladinose sugar (Fig. 1A). These conjugates exhibited higher activity than the parental AZI against clinical isolates of macrolide-resistant *Streptococcus pneumoniae* and vancomycin-resistant *Enterococcus faecium* while showing comparable or even lower activity against other gram-positive strains (Table 1), underscoring the complexity of structure-activity relationships. To validate these observations, we assessed the antibacterial activity of these conjugates against a set of *E. coli* strains with defined macrolide resistance genotypes. All three conjugates failed to inhibit the growth of *E. coli* strains exhibiting the cMLS_B_ resistance phenotype, either due to constitutive *ermC* expression or the A2058G substitution in the 23S rRNA (Table S1). However, AZI-BB conjugates remained moderately active against an *E. coli* strain carrying an inducible *ermCL-ermC* operon, which confers the iMLS_B_ phenotype (Table 2). In this strain, the presence of the inducible *erm* operon caused a 16-fold and 8-fold increase in the minimum inhibitory concentration (MIC) for erythromycin (ERY) and AZI, respectively, compared to the isogenic macrolide-sensitive *E. coli ΔtolC* strain. In contrast, MIC values for chloramphenicol (CHL) and telithromycin (TEL) remained unchanged, consistent with their use as reference agents not affected by inducible *ermC*-mediated resistance (48). Interestingly, the MICs for AZI-BB2 and AZI-BB3 were unaffected by the introduction of the *ermCL-ermC* operon, while AZI-BB1 and an AZI derivative containing an ethylenediamine linker (AZIL) exhibited a 2-fold increase in MICs.

**TABLE 1:** Antibacterial activity of AZI-BB conjugates against a panel of gram-positive strains^*a*^

	Compound, MIC (μg/mL)				
Strain	AZI	AZIL	AZI-BB1	AZI-BB2	AZI-BB3
*Streptococcus pneumoniae* ATCC 49619^*b*^	0.06	0.5	0.125	0.125	0.125
*Streptococcus pneumoniae* B-24 (408)^*c*^, M	4	>32	**1**	**2**	4
*Streptococcus pneumoniae* B-1^*d*^, iMLS_B_	>32	>32	**8**	**8**	**16**
*Streptococcus pneumoniae* 148^*e*^, iMLS_B_	>32	>32	**8**	**8**	**16**
*Streptococcus pneumoniae* 525^*e*^, iMLS_B_	2	16	**1**	2	2
*Streptococcus pneumoniae* 767^*e*^, iMLS_B_	>32	>32	**16**	**16**	**32**
*Streptococcus pyogenes* ATCC 19615^*f*^	≤0.125	nt	0.25	0.25	1
*Staphylococcus aureus* ATCC 29213	1	32	16	16	16
*Staphylococcus aureus* 10^*f*^	1	nt	**0.5**	nt	2
*Staphylococcus epidermidis* ATCC 12228^*f*^	0.5	nt	8	8	32
*Enterococcus faecium* 568^*f*^ , VSE	8	nt	**0.125**	**2**	**0.125**
*Enterococcus faecium* 569^*f*^, VRE	8	nt	**2**	**1**	>32
*Propionibacterium acnes* ATCC 6919^*f*^	≤0.125	nt	≤0.125	0.25	0.5

^*a*^ M: efflux-mediated macrolide resistanse. iMLS_B_: inducible resistance to macrolide, lincosamide, and streptogramin B (MLS_B_) antibiotics. VSE: vancomycin-susceptible *Enterococcus faecium*. VRE: vancomycin-resistant *Enterococcus faecium*. MIC values in bold are lower than those for the parental AZI. At least three replicates of MIC measurements were performed. nt, not tested.

^*b*^ *Streptococcus pneumoniae *ATCC 49619: macrolide-susceptible strain.

^*c*^ *Streptococcus pneumoniae *B-24 (408): clinically isolated macrolide-resistant strain expressing the *mefA* gene.

^*d*^ *Streptococcus pneumoniae *B-1: clinically isolated macrolide-resistant strain expressing the *ermB* gene.

^*e*^ *Streptococcus pneumoniae *148, 525, and 767: clinically isolated macrolide-resistant strains, not characterized.

^*f*^ Antibacterial data are taken from (46).

**TABLE 2:** Antibacterial activity of AZI-BB conjugates against macrolide-sensitive *E. coli ΔtolC* strain and *E. coli ΔtolC* pErmCL-ErmC strain with iMLS_B_ phenotype, expressing an inducible *ermCL-ermC* operon which makes the strain resistant to macrolide antibiotics^*a*^

	Compound, MIC (μM)								
Strain	ERY	AZI	AZIL	AZI-BB1	AZI-BB2	AZI-BB3	BB	TEL	CHL
*E. coli ΔtolC*	4	1	4	4	8	8	64	1	2
*E. coli ΔtolC* pErmCL-ErmC	64	8	8	8	8	8	64	1	2
Fold change of induction (relative to *E. coli ΔtolC*)	16	8	2	2	1	1	1	1	1

^*a*^ At least three replicates of MIC measurements were performed.

These results suggest that the attachment of benzoxaborole moiety through a linker to the AZI scaffold can suppress the induction of *ermC*-mediated resistance, thereby preserving antibacterial activity against iMLS_B_ strains. Notably, the benzoxaborole moiety (BB) alone was inactive against both strains, underscoring the importance of the macrolide scaffold for activity. To determine whether the AZI-BB conjugates inhibit bacterial translation with similar or altered potency compared to parent AZI, we next carried out a series of *in vitro* biochemical assays.

### AZI-BB conjugates are potent inhibitors of protein biosynthesis

Consistent with previous reports, AZI-BB conjugates exhibit strong induction of the pDualrep2 reporter system *in vivo*, suggesting that they likely inhibit protein biosynthesis in bacterial cells, similarly to AZI (Fig. S2) (46). To test this directly, we assessed the ability of AZI-BB compounds to inhibit translation *in vitro* using the PURE system reconstituted from purified *E. coli* translation components. All conjugates, in contrast to BB alone, efficiently inhibited translation (Fig. 1B; Fig. S3), with comparable IC_50_ values across the tested compounds. These findings indicate that incorporation of the benzoxaborole side chain does not significantly affect the translation inhibitory activity.

Among the conjugates, AZI-BB2 displayed the strongest inhibition, surpassing both AZI-BB1, AZI-BB3, and the reference compound AZIL. To further examine how these conjugates interact with the vacant ribosome, we performed a competition-binding assay using BODIPY-labeled ERY (49, 50). All compounds demonstrated a dose-dependent decrease in fluorescence anisotropy (Fig. S4), indicating the ability of the conjugates to displace BODIPY-ERY from its binding site located in the NPET. The apparent dissociation constants (*K′_D_*) derived from these curves were generally similar (Fig. 1C), suggesting that the benzoxaborole moiety neither obstructs proper accommodation of the molecule in the NPET nor substantially alters its affinity. Notably, AZI-BB3 showed a somewhat reduced binding affinity, possibly due to structural differences in its benzoxaborole substituent (Fig. 1A). Despite their efficient ribosome binding and inhibition of translation *in vitro*, AZI-BB conjugates displayed higher MIC values than unmodified AZI against macrolide-susceptible *Staphylococcus* sp. and *E. coli ΔtolC* (Tables 1 and 2), suggesting reduced cellular uptake in gram-negative and some gram-positive strains, which is consistent with earlier findings (46) and likely is due to the conjugates’ increased molecular weight or polarity.

Since the structural modifications made to AZI could result in altered selectivity and the appearance of cytotoxic activity against human cells, we next assessed whether AZI-BB conjugates can inhibit translation in HEK293T whole-cell lysate system at relatively high concentrations (50 μM). In all cases, no translation inhibition was observed (Fig. S5). In addition, AZI-BB2 and AZI-BB3 exhibited favorable cytotoxicity profiles in four human cell lines (MCF7, VA13, A549, and HEK293T) comparable or even superior to those of unmodified AZI (Table S2). AZI-BB1, however, was on average 2.6 times more toxic. These results indicate that AZI-BB2 and AZI-BB3 conjugates retain bacterial selectivity while showing minimal short-term cytotoxic effects on mammalian cells, underscoring their therapeutic potential. To assess whether the conjugates remain active against A2058-methylated ribosomes, we evaluated their activity using *E. coli* 70S ribosomes modified at A2058 by ErmC-methyltransferase (Fig. S6). As expected, all compounds, even at the highest concentration (50 μM), could not fully inhibit translation. However, they still demonstrated ~40% suppression due to the presence of a fraction of unmethylated ribosomes.

Taken together, these data demonstrate that AZI-BB conjugates, similar to parent macrolide AZI, inhibit bacterial translation by binding to the ribosomal NPET. Their activity is similarly affected by A2058-N6-methylation, and they show low levels of cytotoxicity to human cells compared to MIC values against some clinically relevant gram-positive strains (Table 1). These findings support the therapeutic potential of the synthesized AZI-BB conjugates. We next investigated whether these conjugates directly affect the inducible expression of the ErmC methyltransferase.

### AZI-BB conjugates are poor inducers of the *ermC* expression

**Fig 2:**
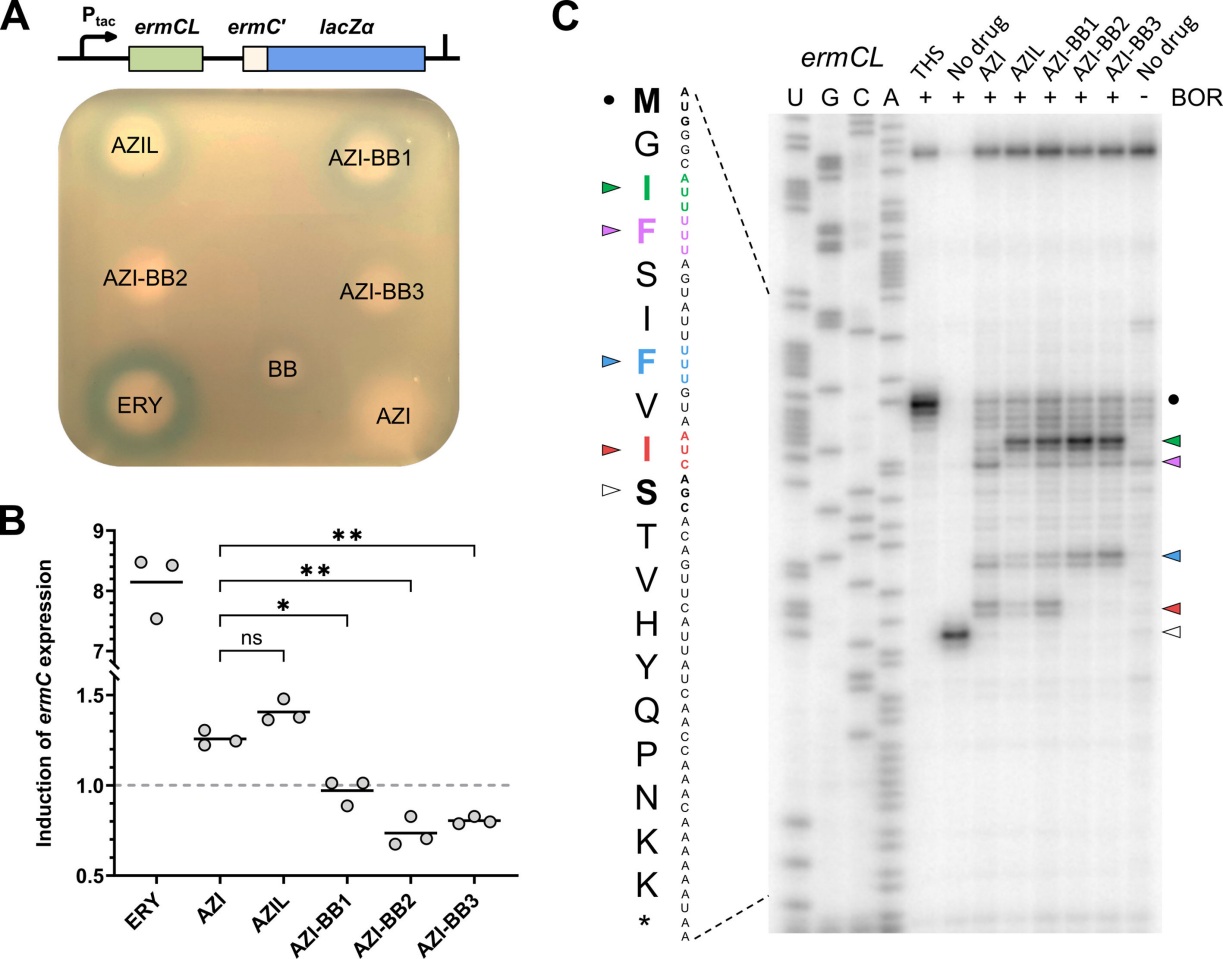
AZI-BB conjugates do not induce translation of *ermC* open reading frame. (**A**) *Top:* Schematic of pERMZα reporter construct. *Bottom:* Agar diffusion assay showing induction of the pERMZα reporter, visualized as blue halos, by various antibacterial compounds. An LB-agar plate containing *E. coli ΔacrB* pERMZα cells was supplemented with IPTG and X-Gal. Solutions of ERY, AZI, AZIL, AZI-BB1-3, and BB were applied to the agar surface. The plate was incubated overnight and photographed. (**B**) Induction of full-length ErmC synthesis upon treatment with AZI derivatives. *In vitro* transcription-translation coupled reactions were carried out in the presence of T7ermCLC(M23L)-FLAG DNA template. All compounds were tested at a final concentration of 1 μM. The intensities of ErmC-FLAG gel bands were quantified and normalized to the untreated control. Corresponding gels are shown in Fig. S8B. Gray dots are individual data points showing induction fold change. Horizontal black lines indicate mean values. The dots located below the gray dashed line correspond to the absence of *ermC* induction. p-values: *P* < 0.01 (*), *P* < 0.001 (**), based on one-way ANOVA with Tukey’s post hoc test; *n* = 3 biologically independent replicates. ns, not significant. (**C**) Toeprinting analysis of AZI derivatives on *ermCL* mRNA in the presence (+) or absence (−) of borrelidin (BOR). The nucleotide and amino acid sequences of the *ermCL* ORF are shown on the left. An asterisk (*) marks the stop codon. Arrowheads indicate toeprint bands corresponding to ribosomes stalled during translation. The codons in the P site of stalled ribosomes are annotated in the mRNA sequence. Ribosome stalling at the start codon (AUG) is marked by black dots. Thiostrepton (THS) was included for translation initiation site mapping. AZI, AZIL, and AZI-BB1-3 were used at 30 μM; THS and BOR at 50 μM.

To assess whether AZI-BB conjugates can induce *ermC* expression, we employed the pERMZα reporter system, which contains the *ermCL-ermC* regulatory cassette with the *ermC* ORF replaced by the *lacZα* gene (48). As expected, ERY strongly activated the reporter, producing a prominent blue halo (Fig. 2A). Among the azithromycin derivatives, AZIL and AZI-BB1 showed weak induction, while AZI-BB3 and especially AZI-BB2 did not activate the reporter. Notably, the absence of a blue halo in response to AZI has been previously discussed and is attributed to drug-specific inhibition of β-galactosidase activity (48), a phenomenon not observed with AZIL. These results are consistent with MIC data from *E. coli ΔtolC* carrying the *ermCL-ermC* operon (Table 2), supporting the conclusion that AZI-BB2 and AZI-BB3 fail to induce full-length ErmC synthesis.

We next examined this phenomenon using a reconstituted *in vitro* translation system. For this, we constructed two sets of DNA templates under a T7 promoter: one encoding the full *ermCL-ermC* operon, and another containing only the *ermC* ORF (Fig. S7A). To enable the detection of ErmC protein by western blot, we tagged its C-terminus with a FLAG tag. Since it was previously noted that the 23^rd^ AUG codon of the *ermC* gene might serve as an alternative translation start site (51), we mutated it to the CUC (Leu) codon, resulting in the T7ermCLC(M23L)-FLAG DNA template. In parallel, the first AUG codon was also mutated to the ACG (Thr) codon, resulting in the T7ermCLC(M1T)-FLAG DNA template. These constructs were validated using a fluorescence-based *in vitro* translation assay with BODIPY-labeled Lys-tRNA^Lys^ in the presence of 1 μM ERY, the optimal concentration reported for induction (51). As expected, ERY induced an ~8-fold increase in the synthesis of full-length ErmC from the *ermCL-ermC* construct, while the shorter isoform starting from the 23^rd^ codon was unaffected by ERY (Fig. 2B; Fig. S7B). Western blot analysis further confirmed these findings (Fig. S7B).

We next examined ErmC induction across a range of AZI concentrations (0.05–2 μM) (Fig. S8A). Translation was fully suppressed at higher AZI concentrations, while lower concentrations were insufficient for induction. Like ERY, AZI induced full-length ErmC synthesis at an optimal concentration of 1 μM. Under these conditions, AZI and AZIL produced modest induction (~1.3-fold and ~1.4-fold, respectively), whereas AZI-BB2 and AZI-BB3 showed no detectable induction (Fig. 2B). These *in vitro* results are in qualitative agreement with the *in vivo* pERMZα reporter data, reinforcing the reliability of the reconstituted translation system for evaluating the induction potential of macrolide derivatives. In summary, AZI-BB conjugates (particularly AZI-BB2 and AZI-BB3) are poor inducers of *ermC* expression, both *in vivo* and *in vitro*. This likely reflects a shift in their context-specific interaction with the translating ribosome, suggesting altered sequence specificity for ribosome stalling and downstream gene regulation.

### AZI-BB conjugates cause premature ribosome stalling

Because *ermC* expression is regulated by site-specific ribosome stalling within the *ermCL* leader ORF, the lack of *ermC* induction by AZI-BB2 and AZI-BB3 (Fig. 2B) could have two possible explanations: (i) these compounds fail to cause ribosome stalling within the *ermCL* coding sequence or (ii) they induce ribosome stalling at an alternative site within *ermCL* coding sequence, which cannot activate translation of the downstream *ermC* ORF. To discriminate between these possibilities, we performed toeprinting analysis using a short *ermCL* mRNA template. To trap ribosomes that might have bypassed the macrolide-sensitive stall site, we added borrelidin (BOR), an inhibitor of threonyl-tRNA synthetase that arrests ribosomes at Thr codons (52). In *ermCL*, the Thr11 codon follows the macrolide-sensitive site, providing a means to assess upstream stalling efficiency.

As expected, AZI caused ribosome stalling at the Phe7 and Ile9 codons when they are placed in the P site of the ribosome (Fig. 2C). However, only stalling at the Ile9 codon is required for induction of ErmC synthesis, as it is in the case of ERY (51, 53). AZIL and AZI-BB1, in addition to stalling at Phe7 and Ile9, also caused premature arrest at Ile3. In contrast, AZI-BB2 and AZI-BB3 completely lacked toeprinting bands at Ile9 and instead showed enhanced stalling at earlier sites, notably Ile3 and Phe7. This indicates that AZI-BB2 and AZI-BB3 trigger ribosome arrest upstream of the canonical macrolide-sensitive site, thereby preventing the mRNA rearrangement required to trigger *ermC* expression. In addition, no stalling at the Ser10 codon was observed, despite the presence of BOR, indicating that 100% of the ribosomes were arrested upstream during translation.

To investigate whether AZI-BB-induced stalling depends on specific mRNA sequence contexts, we extended the toeprinting assay to other regulatory leader sequences: *ermBL* and *ermDL*, which control the expression of *ermB* and *ermD*, respectively. In both cases, AZI-BB2 and AZI-BB3 induced premature stalling at Val3 in *ermBL* and Met1 in *ermDL* (Fig. S9). Additionally, the downstream macrolide-sensitive sites normally targeted by AZI exhibited reduced ribosome occupancy in the presence of AZI-BB2 and AZI-BB3. These results suggest that AZI-BB conjugates may similarly impair *erm*-type regulation in other iMLS_B_ pathogens harboring the *ermBL-ermB* or *ermDL-ermD* operons, since their expression is regulated in a similar manner as that of *ermCL-ermC*.

**Fig 3:**
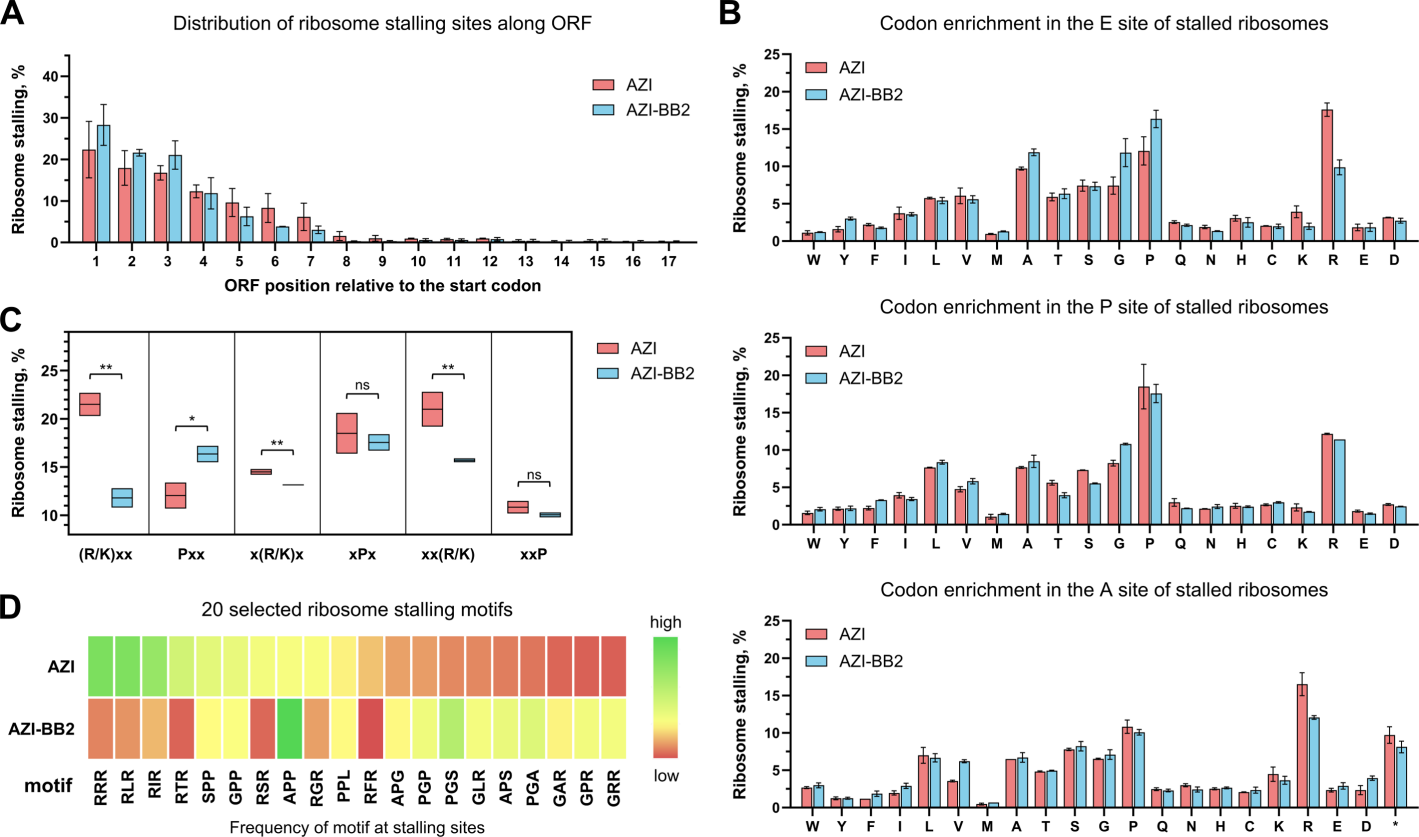
Toe-seq analysis reveals altered context specificity of AZI-BB2 compared to AZI. (**A**) Distribution of ribosome stalling sites along the coding region of mRNAs. Error bars represent standard deviation across replicates. (**B**) Enrichment of codons occupying E, P, and A sites of ribosomes stalled in the presence of AZI or AZI-BB2. Enrichment is shown as the relative frequency of encoded amino acids, normalized using *MaxStopProbability* scores. An asterisk (*) denotes stop codons. Only codon positions 3–10 of the ORF are included in the analysis, as they represent the variable region of mRNA. Error bars indicate standard deviation. (**C**) “Low-medium-high” bar chart illustrating the relative occurrence of stalling motifs associated with AZI in AZI- *vs*. AZI-BB2-treated samples. Horizontal black lines mark the mean. p-values: *P* < 0.1 (*), *P* < 0.05 (**), based on a one-tailed unpaired two-sample *t*-test; *n* = 2 independent replicates. ns, not significant. (**D**) Heatmap color scale indicates the frequency of ribosome stalling motifs detected by Toe-seq and adjusted using *MaxStopProbability* scores. The 10 most enriched motifs from AZI-treated samples were combined with the top 10 motifs enriched in AZI-BB2-treated samples for comparative analysis.

Since the conventional toeprinting analysis cannot reveal any substantial context specificity of AZI-BBs action, we selected AZI-BB2 (the most promising conjugate) for analysis by Toe-seq, a recently developed method to assess context specificity of translation inhibitors in a multiple parallel assay (47). Similar to ribosome profiling, Toe-seq provides a snapshot of ribosomal movement along mRNAs under given conditions. However, it utilizes a library of short artificial DNA templates containing a randomized 30-nt-long region in the coding sequence, instead of native cellular mRNAs. Toe-seq represents a high-throughput version of toeprinting analysis and generates digital toeprints for each mRNA in the *in vitro* expressed library. Toe-seq was performed with 50 μM AZI or AZI-BB2 in duplicates, along with untreated controls. Data sets for antibiotic-treated samples were normalized to the controls and filtered according to a set of criteria (see Materials and Methods for details). The calculated *MaxStopProbability* scores, representing the relative efficiency of antibiotic-induced ribosome stalling, were well correlated between both pairs of replicates (Fig. S10). We first compared the positional distribution of ribosome stalls across coding regions. AZI-BB2-treated samples showed a strong bias for stalling near the 5′ end of the coding sequence, whereas AZI-treated samples exhibited a more uniform distribution along the mRNA (Fig. 3A). This confirms and generalizes our earlier observations from toeprinting experiments (Fig. 2C; Fig. S9).

Next, we analyzed whether specific amino acids are associated with the sites of most prominent ribosome stalling induced by treatment with AZI or AZI-BB2. Therefore, we looked for a possible enrichment of specific amino acids corresponding to the codons located in E, P, and A sites of arrested ribosomes. Overall, the context specificity of AZI-BB2 resembled that of AZI (Fig. 3B; Fig. S11), but with notable differences. AZI-BB2 was significantly less likely to induce stalling at the (R/K)xx and xx(R/K) motifs, registering 11.8% and 15.7% of stalling events, respectively, compared to AZI, which showed 21.5% and 21.0% for the same motifs (Fig. 3C). Moreover, AZI-BB2 showed a ~5-fold reduction in stalling at the canonical (R/K)x(R/K) motif: 9.78% for AZI *vs*. only 1.95% for AZI-BB2 (Fig. S12A)—the most dramatic difference between the two compounds. This suggests reduced sensitivity of AZI-BB2 to positively charged residues flanking the stall site.

In contrast, both antibiotics exhibited similar stalling efficiencies at xPx and xPP motifs (Fig. 3C; Fig. S12A). However, when comparing the 20 most enriched stalling motifs, 10 each from AZI and AZI-BB2 data sets, AZI-BB2 was more likely to stall at the APP motif while being less likely to target different (R/K)x(R/K) motifs (Fig. 3D; Fig. S12B). It also showed increased stalling at PGx and Gx(R/K) motifs (Fig. S12A). Additional minor motif preferences unique to AZI-BB2 are summarized in Fig. S13 to S15.

Taken together, the Toe-seq results suggest that AZI-BB2 differs from AZI in two key ways: (i) it preferentially induces ribosome stalling near the beginning of coding sequences, especially within the first four codons, and (ii) it exhibits altered sequence specificity, with reduced dependence on motifs enriched in *erm* leader sequences. These properties likely explain the inability of AZI-BB2 to activate *ermC* expression and support its potential for bypassing inducible macrolide resistance. The tendency of AZI-BBs to cause premature stalling might be driven by steric clashes between the nascent peptide and the modified macrolide scaffold, especially during early elongation stages.

### AZI-BB conjugates form additional interactions with 23S rRNA

**Fig 4:**
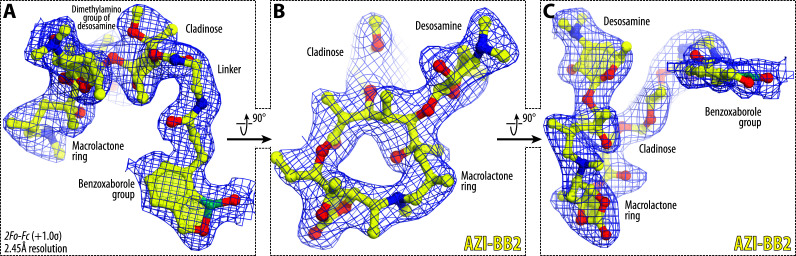
Electron density maps of ribosome-bound AZI-BB2. (**A–C**) *2Fo-Fc* Fourier electron density maps showing AZI-BB2 (yellow) in complex with the *T. thermophilus* 70S ribosome (blue mesh), presented in three mutually perpendicular views. The refined model of ribosome-bound AZI-BB2 is overlaid with its corresponding electron density map, contoured at 1.0σ. Carbon atoms are shown in yellow, nitrogens are in blue, oxygens are in red, and boron is colored teal. Note that the benzoxaborole side chain is clearly resolved, allowing unambiguous placement of this moiety within the electron density map.

**Fig 5:**
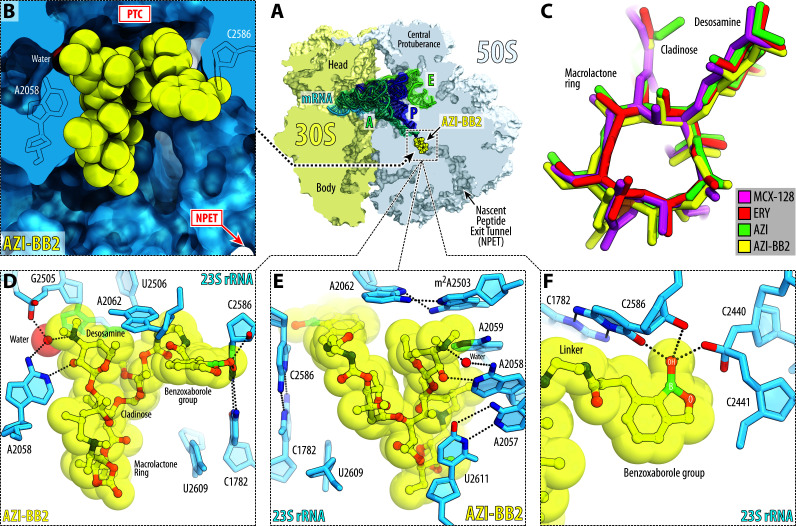
Structure of AZI-BB2 in complex with the wild-type *T. thermophilus* 70S ribosome. (**A**) Overview of the binding site of AZI-BB2 (yellow) in the 70S ribosome, viewed as a cross-cut section through the nascent peptide exit tunnel (NPET). The 30S subunit is shown in light yellow, the 50S subunit is in light blue, and the mRNA, A-, P-, and E-site tRNAs are colored blue, teal, navy blue, and green, respectively. (**B**) Close-up view of AZI-BB2 bound in the NPET, highlighting the characteristic interactions with the two opposite walls of the NPET: one with the surface formed by nucleotides A2058 and A2059 of the 23S rRNA (*E. coli* numbering) and the second one with the nucleotide C2586. (**C**) Comparison of the structures of ribosome-bound AZI-BB2 (yellow) with the previous structures of AZI (green, PDB entry 8FC2 [54]), ERY (red, PDB entry 6XHX [7]), or macrolone MCX-128 (magenta, PDB entry 8VTW [39]). All structures were aligned based on domain V of the 23S rRNA. (**D–F**) Close-up views of AZI-BB2 in the NPET of the 70S ribosome, highlighting their H-bond (dashed lines) interactions with the nucleotides of the 23S rRNA.

To gain deeper insight into the binding mode of AZI-BB2 and understand how its benzoxaborole moiety engages the ribosome, we determined the high-resolution X-ray crystal structure of the *Thermus thermophilus* 70S ribosome in complex with AZI-BB2. The structure was solved at 2.45 Å resolution (Table S3), and the resulting electron density map revealed well-defined features corresponding to all chemical groups of AZI-BB2, enabling unambiguous placement of the compound within the ribosome (Fig. 4). The structure also contains non-hydrolyzable aminoacylated Phe-tRNA^Phe^ and fMet-tRNA_i_^fMet^ in the A and P sites, respectively. Although the complex does not include a peptidyl-tRNA bearing a stalling peptide, it nonetheless captures the ribosome in a functional pre-peptidyl transfer state of the PTC, with substrates in both the A and P sites correctly positioned for nucleophilic attack (Fig. 5A).

As expected, the macrolactone ring and desosamine sugar of AZI-BB2 occupy the canonical macrolide-binding pocket within the NPET (Fig. 5B). The binding pose of the macrolide moiety closely resembles that observed for unmodified AZI and other classic macrolides, such as ERY, or macrolones (Fig. 5C). Importantly, the desosamine sugar of AZI-BB2 forms hallmark interactions with the ribosome: a direct hydrogen bond with the N1 atom of A2058, and an additional water-mediated hydrogen bond with its N6 atom—contacts that are well-established anchors for macrolide binding (Fig. 5D and E).

In addition to the conserved interactions of the macrolide core, strong and continuous electron density was observed extending from the 4″-hydroxy position of the cladinose sugar, corresponding to the benzoxaborole moiety tethered via a flexible linker (Fig. 4). Interestingly, the aromatic ring of the AZI-BB2 side chain does not participate in π-π stacking interactions as might be expected for a flat aromatic system. Instead, it forms at least two hydrogen bonds (H-bonds) with the 23S rRNA nucleotides C2441 and C2586 (Fig. 5E and F). Strikingly, the benzoxaborole engages the NPET wall at an almost perpendicular angle (Fig. 5F), contrasting sharply with the side chains of other macrolide derivatives, such as the alkyl-aryl moieties of ketolides or the fluoroquinolone extensions of macrolones, which typically approach the tunnel wall in a more parallel orientation and rely on π-π stacking. Despite these additional H-bonds, the local rRNA conformation within the macrolide-binding site remains unperturbed, suggesting that the benzoxaborole extension does not distort the canonical drug-binding architecture. It is therefore intriguing that the apparent binding affinity of AZI-BB2 for the ribosome remains similar to that of unmodified AZI (Fig. 1C), despite its capacity to form new stabilizing interactions with NPET.

Taken together, our structural data demonstrate that AZI-BB2 retains all critical macrolide-ribosome contacts while introducing new interactions via its benzoxaborole extension. These additional contacts likely perturb nascent peptide progression through the NPET in a manner distinct from AZI, contributing to the altered stalling behavior observed in toeprinting and Toe-seq experiments. The well-defined density and specific orientation of the benzoxaborole moiety underscore its potential as a modular pharmacophore for rationally tuning antibiotic-ribosome interactions in a predictable and structure-guided fashion.

## DISCUSSION

In this study, we compared the mechanism of action of three azithromycin-benzoxaborole conjugates (AZI-BB1-3) with that of the parent compound AZI and its ethylenediamine-linked derivative (AZIL) (Fig. 1A). Our data demonstrate that, although AZI-BB conjugates, similar to AZI, inhibit bacterial translation by binding to the ribosomal NPET, they retain activity against a model *E. coli* strain carrying an inducible *ermCL-ermC* operon (Table 2). In both *in vitro* and *in vivo* assays, AZI-BB2 and AZI-BB3 failed to induce full-length ErmC synthesis, while AZI and AZIL induced only modest expression (Fig. 2A and B).

Somewhat surprisingly, even AZI itself exhibited only a 1.3-fold increase in ErmC production *in vitro*, substantially lower than the 8-fold increase observed with ERY. This was unexpected since AZI is usually considered a strong inducer of the iMLS_B_ phenotype, resulting in an average 128-fold increase in MIC values measured among gram-positive clinical isolates (34, 37, 55). Nevertheless, some *erm*-type regulation systems might be less sensitive to semisynthetic AZI compared to natural ERY, possibly due to the narrower context specificity of AZI relative to ERY. In this regard, AZI-based conjugates may have more therapeutic potential than ERY-based ones, owing to their inherent reduced propensity to induce expression of certain *erm* genes.

Our further toeprinting and Toe-seq experiments provided a mechanistic explanation for the resistance-evasion properties of AZI-BB conjugates. Increased probability to cause ribosome stalling closer to the 5′-end of coding sequences (especially within the first four codons), along with reduced specificity to classic macrolide-stalling motifs, likely underlies the inability of AZI-BB2 and AZI-BB3 to activate *ermC* expression.

The trends observed for AZI-BB conjugates closely parallel findings from our previous work on AZI conjugates with chloramphenicol and metronidazole (AZI-CHL and AZI-MNZ, respectively) (56). These compounds, which also feature substituents attached via a linker to the C-4″ position of AZI, showed similarly weak *ermC* induction and premature ribosome stalling on the *ermCL* template. In fact, the stalling patterns induced by AZI-CHL and AZI-MNZ were strikingly similar to those caused by AZI-BB2 and AZI-BB3, suggesting a common mechanism whereby C-4″-linked extensions alter the positioning of the nascent chain in the NPET and shift drug-ribosome interactions upstream.

However, subtle differences in stalling patterns and context specificity suggest that the nature of the attached moiety still plays a role. For instance, some macrolones, macrolide-quinolone hybrids that inhibit both the ribosome and DNA gyrase, also exhibit reduced propensity to stall at classic macrolide-sensitive motifs and exhibit poor induction of *erm*-type reporters (39). However, the specific distribution of stalling sites for macrolones differed from those observed here for AZI-BB conjugates, highlighting that context specificity of ribosome stalling is finely tuned by the chemical identity of the side chain attached to the macrolactone ring.

Our structural analysis shed light on how AZI-BB2 interacts with the ribosome, revealing additional H-bonds formed between the benzoxaborole moiety and nucleotides C2441 and C2586 of the 23S rRNA (Fig. 5). These interactions differ from those previously reported for ketolide and macrolone side chains and likely create a steric clash with the emerging nascent peptide, explaining the observed premature stalling. In line with recent structural studies on macrolones (38, 39), our findings underscore how ribosome-bound moieties extending into the NPET can introduce novel interaction networks that reshape translation arrest landscapes.

It would be fair to note that while AZI-BB conjugates, especially AZI-BB1 and AZI-BB2, exhibited higher activity than AZI against macrolide-resistant *Streptococcus pneumoniae* and vancomycin-resistant *Enterococcus faecium*, they demonstrated considerably lower activity against some other bacterial strains, including *Staphylococcus* sp. and *E. coli* (Tables 1 and 2), underscoring the complexity of structure-activity relationships. Further optimization of AZI-BB side chains guided by structural insights may expand the spectrum of activity, leading to the development of next-generation ribosome-targeting antibiotics capable of evading inducible resistance to macrolides.

### Conclusions

Our study uncovers how chemical modification of AZI with benzoxaborole moieties alters its interaction with the ribosome and modulates its mechanism of action. We found that AZI-BB conjugates (AZI-BB1-3) efficiently inhibit bacterial translation and maintain binding affinity to the ribosome comparable to unmodified AZI. Remarkably, these compounds retain activity against the *E. coli* strain carrying the inducible *ermCL-ermC* operon, a known mechanism of macrolide resistance. Mechanistically, this resistance-bypassing effect is attributed to the inability of AZI-BB2 and AZI-BB3 to induce *ermC* expression, likely due to altered sequence specificity of ribosome stalling. Structural analysis of the AZI-BB2/ribosome complex revealed that the benzoxaborole extension forms additional H-bonds with conserved 23S rRNA nucleotides in the NPET, introducing new interactions without disrupting the canonical macrolide-binding site. Overall, this study expands our understanding of how structural modifications at the C-4″ position of AZI influence drug-ribosome interactions and translation arrest specificity. Our findings support a rational, structure-guided approach to designing macrolide-based antibiotics with enhanced ability to overcome *erm*-mediated resistance.

## MATERIALS AND METHODS

### Chemical compounds

Azithromycin-benzoxaborole conjugates (AZI-BB1, AZI-BB2, and AZI-BB3) as well as azithromycin derivative with the ethylenediamine linker (AZIL, 4″-*O*-((2-aminoethyl)carbamoyl)−11,12-cyclic carbonate of azithromycin) were synthesized as described previously (46, 56). Azithromycin (AZI), benzoxaborole derivative (BB, also known as AN3661 [44]), erythromycin (ERY), telithromycin (TEL), chloramphenicol (CHL), thiostrepton (THS), borrelidin (BOR), cycloheximide (CHX), and levofloxacin (LEV) were purchased from Sigma-Aldrich, USA.

The purity of the examined compounds was monitored with the LC-MS analysis using the Acquity UPLC System (Waters, USA) supplied with the Acquity UPLC BEH C18 column (1.7 μm, 2.1 × 50 mm) maintained at 35°C and coupled to a diode array detector PDA and triple quadrupole mass spectrometer Xevo TQD (Waters, USA) with an ESI ionization source. The MS1 parameters were as follows: polarity ES+, desolvation gas flow of 800 L/h, cone gas flow of 50 L/h, source temperature of 120°C, desolvation temperature of 450°C, capillary voltage of 3.0 kV, cone voltage of 55 V, mass range 100–2,000 Da. The mobile phases were A (20 mM formic acid in water) and B (20 mM formic acid in acetonitrile). The LC parameters were as follows: flow rate of 0.5 mL/min and gradient (0–3.0 min from 5% to 100% B, 3.0–4.0 min with 100% B, 4.0–4.1 min from 100% to 5% B, and 4.1–5.0 min with 5% B).

### Plasmids and cloning

Plasmids pERMCTP (57) (referred to here as pErmC) and pERMZα (48) were kindly provided by Dr. Alexander S. Mankin, University of Illinois, USA. The pDualrep2 plasmid was constructed previously as described in (58).

Recently published construct pErmCL-ErmC (56) was slightly modified in order to obtain a more native sequence of the gene. For this purpose, the vector backbone was amplified using 5′-phosphorylated primers AflII-del-F and AflII-del-R (Table S4), followed by blunt-end ligation. The created construct is referred to hereafter as just pErmCL-ErmC.

The plasmid pErmCL-ErmC-Flag, with the FLAG tag being fused to the C-terminus of the ErmC via the GGGS linker, was obtained by PCR amplification using 5′-phosphorylated primers ErmC-Flag-C-F and ErmC-GGGS-C-R (Table S4), and the pErmCL-ErmC plasmid as a template, followed by blunt-end ligation.

In order to differentiate between two ErmC isoforms on a gel, we created two constructs harboring mutations at the first and 23^rd^ AUG codons. The construct pErmCL-ErmC(M1T)-Flag was obtained using primers AflII-del-F and 1ATG>ACG-R, whereas pErmCL-ErmC(M23L)-Flag was obtained with primers 23ATG-exch-F and 23ATG>CTC-R (Table S4). In both cases, the primers were 5′-phosphorylated, pErmCL-ErmC-Flag was used as a template, and the PCR products were subjected to blunt-end ligation.

Sequences of intermediate products and final constructs were confirmed by Sanger sequencing.

### Bacterial strains and cultivation conditions

The *E. coli* JW5503 strain with the deletion of the *tolC* gene was kindly provided by Prof. Hironori Niki, National Institute of Genetics, Japan (59). The kanamycin resistance cassette was further removed from this strain according to the procedure described in a previous study (60). The obtained strain (referred to here as *E. coli ΔtolC*) was transformed with pErmC, pErmCL-ErmC, or pDualrep2 plasmids. *E. coli* SQ171 strains with the deletion of all seven rRNA operons and the *tolC* gene (referred to here as *E. coli* SQ171 *ΔtolC*) and transformed with the pAM552 plasmid encoding either native (WT) or mutated (A2058G) *rrnB* operon were obtained as described previously (61). The *E. coli* MRE600 strain was kindly provided by Prof. Andrey L. Konevega, Peter the Great St. Petersburg Polytechnic University, Russia (62, 63). This strain was transformed with the pErmC plasmid. The *E. coli* JM109 strain with the deletion of the *acrB* gene (referred to here as *E. coli ΔacrB*) was kindly provided by Dr. Alexander S. Mankin (57). This strain was transformed with the pERMZα plasmid. The *E. coli* JM109 strain was used for DNA cloning (64). All *E. coli* strains were cultivated at 37°C in Miller’s Luria–Bertani (LB) medium supplied with 100 μg/mL ampicillin, if required.

Gram-positive bacterial strains were from American Type Culture Collection (ATCC) or clinical isolates from the collection of Gause Institute of New Antibiotics. Clinical isolates of *Streptococcus pneumoniae* were kindly provided by Prof. Sergey V. Sidorenko, Federal State-Financed Institution Pediatric Research and Clinical Center for Infectious Diseases under the Federal Medical Biological Agency, Russia. All Gram-positive strains were cultivated at 36°C in cation-corrected Mueller–Hinton II broth (BD BBL, France) supplied with 5% lysed sheep blood and 20 mg/L β-NAD (Hem Ltd., Russia), essential for the growth of *S. pneumoniae*.

### Minimum inhibitory concentration (MIC) assay

For *E. coli* strains, MIC values were determined using the liquid broth microdilution assay in 96-well sterile plates and a total volume of 200 μL per well, as described in (65), with minor modifications indicated below. Initially, all compounds were dissolved in dimethyl sulfoxide (DMSO) to achieve a concentration of 20 mM. Then they were diluted with fresh LB medium supplemented with 100 μg/mL ampicillin, if required. Subsequently, 2-fold serial dilutions were performed and *E. coli* cultures in the log growth phase diluted 1:100 were added to each well. The plates were incubated overnight (16–20 h) at 37°C with constant shaking at 200 rpm (Shaker-Incubator ES-20/80, BioSan, Latvia). Cell growth was assessed by registering the OD_600_ with the VICTOR X5 Multilabel Plate Reader (PerkinElmer, USA). MIC was defined as the lowest concentration of a chemical compound that inhibits the visible growth of the bacterial strain. At least three replicates were performed.

For gram-positive strains, MIC values were determined exactly as described in (46).

### Agar diffusion assay using reporter strains

Reporter strains *E. coli ΔtolC* pDualrep2 and *E. coli ΔacrB* pERMZα were used as described previously (48, 58). Briefly, the overnight culture of *E. coli ΔtolC* pDualrep2 was plated on 1.5% LB-agar solid medium supplied with 100 μg/mL ampicillin and left to dry for a while until the next step. The overnight culture of *E. coli ΔacrB* pERMZα was diluted 1:100 in liquid warm (45°C–50°C) 1.5% LB-agar medium supplied with 100 μg/mL ampicillin, 0.5 mM IPTG, and 80 μg/mL X-Gal. After a brief mixing, the cell suspension was poured into a sterile Petri dish and left to solidify. Appropriate solutions of chemical compounds (26.7 nmol of ERY, AZI, TEL, AZIL, BB, AZI-BB1, AZI-BB2, AZI-BB3, and 0.1 nmol of LEV) were placed on the surface of dried agar plates containing a reporter strain. Plates were incubated overnight at 37°C and either photographed on camera or scanned with the ChemiDoc Imaging System (Bio-Rad Laboratories, USA) using two channels: “Cy3-blot” (emission filter 605 ± 50 nm, green pseudocolor) for TurboRFP fluorescence and “Cy5-blot” (emission filter 695 ± 50 nm, red pseudocolor) for Katushka2S fluorescence. Images were analyzed and visualized using the Image Lab software (version 6.0.1) (Bio-Rad Laboratories, USA).

### *In vitro* translation assays

#### *In vitro* translation in a bacterial cell-free system

Firefly luciferase (Fluc) mRNA was prepared using the MEGAscript T7 Transcription Kit (Thermo Fisher Scientific, USA). Translation reactions (3 μL total volume) were carried out with the PURExpress *in vitro* Protein Synthesis Kit (New England Biolabs, USA) according to the manufacturer’s protocol. Each reaction was supplied with 2 U of RiboLock RNase Inhibitor (Thermo Fisher Scientific, USA), 0.17 mM of D-luciferin (Sigma-Aldrich, USA), 60 ng of Fluc mRNA, and antibiotic solution. Control reactions were supplemented with nuclease-free water instead of the antibiotic solution. Assembled reactions lacking mRNA were pre-incubated at room temperature (RT) for 5 min. Translation was initiated by the addition of mRNA, and reaction mixtures were immediately transferred into the 384-well white microplate (Corning, USA). Chemiluminescence was continuously registered using the VICTOR X5 Multilabel Plate Reader (PerkinElmer, USA) at 37°C for 1 h. Maximum Fluc accumulation rates were calculated for each sample and normalized to a positive control (nuclease-free water). The IC_50_ values of translation inhibition were calculated and visualized using the GraphPad Prism software (version 10.1.2).

#### *In vitro* translation in a mammalian cell-free system

Whole-cell extract was prepared from the HEK293T cell line as described previously (66) with minor modifications: harvested cells were not treated with lysolecithin buffer. Instead, they were immediately suspended in an equal volume of ice-cold hypotonic extraction buffer [20 mM HEPES-KOH (pH 7.5), 10 mM CH_3_COOK, 1 mM Mg(CH_3_COO)_2_, 4 mM DTT, and cOmplete EDTA-free Protease Inhibitor Cocktail (Roche, Switzerland)], incubated for 5 min on ice and disrupted in a tiny Dounce homogenizer by 20–25 strokes. The lysate was clarified by centrifugation for 10 min at 10,000 × *g* at 4°C. Aliquots were frozen in liquid nitrogen and stored at −80°C.

Translation reactions were carried out exactly as described in (67). Reactions supplemented with nuclease-free water instead of the antibiotic solution were used as positive controls. Translation efficiencies were calculated and visualized using the GraphPad Prism software (version 10.1.2).

#### *In vitro* translation in a Δ ribosome system

Translation reactions (5 μL total volume) were carried out with the PURExpress Δ Ribosome Kit (New England Biolabs, USA) according to the manufacturer’s protocol. Each reaction was supplied with 0.9 μL of 3.1 μM purified ribosomes in aqueous solution, 2 U of RiboLock RNase Inhibitor (Thermo Fisher Scientific, USA), 50 ng of Fluc mRNA, and antibiotic solution. Control reactions were supplemented with nuclease-free water instead of the antibiotic solution. Assembled reactions lacking mRNA were pre-incubated at RT for 5 min and then placed back on ice. After mRNA addition, reaction mixtures were incubated at 37°C for 30 min. Then, 5 μL of the Bright-Glo Luciferase Assay Substrate (Promega, USA) was added to the reaction mixtures, and they were immediately transferred into the 384-well white microplate (Corning, USA). The relative quantity of accumulated Fluc was estimated by measuring chemiluminescence using the VICTOR X5 Multilabel Plate Reader (PerkinElmer, USA). The values were normalized to a positive control (nuclease-free water) and visualized using the GraphPad Prism software (version 10.1.2).

### *In vitro* competition-binding assay

Affinity of compounds to the *E. coli* 70S ribosomes was analyzed with a competition-binding assay based on fluorescence anisotropy as described previously (49, 50). The ribosomes were purified from the *E. coli* MRE600 strain according to a published procedure (68) and pre-incubated for 15 min at 37°C in binding buffer [20 mM HEPES-KOH (pH 7.5), 50 mM NH_4_Cl, 10 mM Mg(CH_3_COO)_2_, 4 mM β-mercaptoethanol, 0.05% (vol/vol) Tween-20] before the experiment. Fluorescently labeled erythromycin (BODIPY-ERY, 16 nM) was mixed with the ribosomes (40 nM) in the buffer and incubated for 30 min at RT. Then, solutions of the tested compounds were added to the obtained complexes to final concentrations from 1 to 500 nM and incubated for 2 h at RT in a 384-well plate. The values of fluorescence anisotropy were measured using the VICTOR X5 Multilabel Plate Reader (PerkinElmer, USA). Filters of 485 and 535 nm were used for excitation and emission, respectively. To calculate the apparent dissociation constants (*K′_D_*), the assumption that the competitive equilibrium binding of two ligands occurs at a single binding site was used (69). The apparent dissociation constant of the BODIPY-ERY-ribosome complex was assumed to be 16 nM. For each compound, four replicates were performed. Values of apparent dissociation constants (*K′_D_*) are given as means with 95% confidence intervals. The data were processed and visualized using GraphPad Prism software (version 10.1.2).

### Ribosome purification

Overnight culture of the *E. coli* MRE600 pErmC strain was diluted 1:100 in 300 mL of fresh LB medium supplied with 100 μg/mL ampicillin and 100 μg/mL erythromycin and incubated at 37°C with constant shaking (180 rpm, Innova 44 Shaker, New Brunswick Scientific, USA) to an OD_600_ of 0.6–0.8. Cells were cooled on ice, harvested by centrifugation for 15 min at 7,000 rpm at 4°C (centrifuge J2-HS, rotor JA-10, Beckman Coulter, USA), washed with 30 mL of ice-cold PBS (10% of initial volume), and centrifuged for 15 min at 1,500 × *g* at 4°C. Cell pellets were frozen in liquid nitrogen and stored at −80°C before proceeding to the next step. Then, the cells were thawed on ice and resuspended in ice-cold lysis buffer (10 mM Tris-OAc [pH 8.2], 14 mM Mg(OAc)_2_, 60 mM KCl, 1 mM DTT), with 1 mL of lysis buffer being added for each 1 g of cells. The suspension was mixed with acid-washed glass beads (particle size ≤ 106 μm, Sigma-Aldrich, USA), with 1 g of beads being used for each 1 g of cells, and subjected to vigorous vortexing for 1 h at 4°C to disrupt the cells. Resulting lysates were centrifuged for 15 min at 16,000 × *g* at 4°C. The supernatant was additionally clarified by centrifugation for 70 min at 30,000 × *g* at 4°C (Optima MAX-XP Ultracentrifuge, MLA-130 rotor, Beckman Coulter, USA). Subsequently, *E. coli* ribosomes were precipitated from the obtained S30 extract through a sucrose cushion, as described below. The supernatant (~ 1 mL) was loaded slowly over 5 mL of 10% sucrose lysis buffer (10 mM Tris-OAc [pH 8.2], 14 mM Mg(OAc)_2_, 60 mM KCl, 1 mM DTT, 10% [wt/vol] sucrose), forming a layer, and centrifuged for 100 min at 110,000 × *g* at 4°C (Optima MAX-XP Ultracentrifuge, MLA-80 rotor, Beckman Coulter, USA). Thereafter, the supernatant was discarded, and the pellet was dissolved overnight in 200 μL of the same ice-cold lysis buffer without sucrose. The resulting solution was aliquoted, frozen in liquid nitrogen, and stored at −80°C.

### Human cell lines and cultivation conditions

Human breast cancer cell line MCF7 and human lung adenocarcinoma cell line A549 were kindly provided by Dr. Sergey E. Dmitriev, A.N. Belozersky Institute of Physico-Chemical Biology, Lomonosov Moscow State University, Russia. Immortalized human lung fibroblast cell line VA13 was kindly provided by Dr. Maria P. Rubtsova, Department of Chemistry, Lomonosov Moscow State University, Russia. Human embryonic kidney 293T (HEK293T) cell line was kindly provided by Dr. Ekaterina S. Knyazhanskaya, Lomonosov Moscow State University, Russia.

MCF7, VA13, A549, and HEK293T cell lines were maintained in DMEM/F-12 (Servicebio, China) culture medium containing 2 mM L-alanyl-L-glutamine (GlutaMAX Supplement, Thermo Fisher Scientific, USA), 10% (vol/vol) fetal bovine serum (Capricorn Scientific, Germany), 50 U/mL penicillin, and 50 μg/mL streptomycin (Thermo Fisher Scientific, USA) at 37°C in the humidified incubator MCO-18AC (Sanyo, Japan) supplied with 5% CO_2_. All cell cultures were routinely tested for mycoplasma (MycoReport Kit, Evrogen, Russia).

### *In vitro* survival assay (MTT assay)

The cytotoxicity of the compounds was measured using the MTT assay first reported by T. Mossman (70) with minor modifications. A total of 2,500 cells per well for MCF7 and HEK293T cell lines or 3,000 cells per well for A549 and VA13 cell lines were plated out in 135 µL of DMEM/F-12 media (Servicebio, China) in a 96-well plate and incubated at 37°C in the 5% CO_2_ incubator for 20 h without treatment. Then, the tested compounds were diluted in culture medium to the desired final concentrations such that the final DMSO concentration never exceeded 0.5%. Resulting solutions (15 µL) were added to the cells to final concentrations from 45 nM to 100 μM (eight dilutions), and the plates were incubated for 72 h at 37°C in the 5% CO_2_ incubator. Thereafter, the MTT reagent (3-(4,5-dimethylthiazol-2-yl)2,5-diphenyl tetrazolium bromide, PanEco, Russia) dissolved in PBS was added to each well to a final concentration of 0.5 g/L, and the plates were additionally incubated for 2 h at 37°C in the 5% CO_2_ incubator. Then, the solution containing the MTT reagent was discarded, and 140 μL of DMSO (PharmaMed LLC, Russia) was added. The plates were placed on a shaker (120 rpm) for 15–20 min in order to dissolve the dye (formazan), and its concentration was measured using the VICTOR X5 Multilabel Plate Reader (PerkinElmer, USA) at 565 nm. At least three experimental replicates were performed. The resulting data were used to calculate dose-response dependencies and cytotoxic concentrations (CC_50_) indicating the concentration required to reduce cell viability by 50% in comparison with untreated cells. The data were processed and visualized using the GraphPad Prism software (version 10.1.2).

### *In vitro* protein synthesis assay

The induction of the ErmC protein synthesis *in vitro* was monitored by the following assay. First, a set of DNA constructs was prepared by PCR using primers T7ermCLC-fwn and T7ermCLC-rvn (Table S4) in order to obtain T7ermCLC constructs harboring a T7 promoter and the whole *ermCL-ermC* operon. Primers T7ermC-fwn and T7ermCLC-rvn (Table S4) were used to generate a control set of T7ermC constructs containing only the *ermC* gene under the control of the T7 promoter. In both cases, the following plasmids were used as PCR templates: pErmCL-ErmC, pErmCL-ErmC-Flag, pErmCL-ErmC(M1T)-Flag, and pErmCL-ErmC(M23L)-Flag.

Resulting DNA constructs were expressed in a bacterial transcription-translation coupled system. Briefly, translation reactions (3 μL total volume) were carried out with the PURExpress *In vitro* Protein Synthesis Kit (New England Biolabs, USA) according to the manufacturer’s protocol. Each reaction was supplied with 2 U of RiboLock RNase Inhibitor (Thermo Fisher Scientific, USA), 0.1 μL of fluorescently labeled Lys-tRNA^Lys^ (FluoroTect Green_Lys_ *in vitro* Translation Labeling System, Promega, USA), 20 ng of T7ermCLC or T7ermC DNA template, and antibiotic solution. Control reactions were supplemented with nuclease-free water instead of antibiotic solution. Before the addition of templates, reaction tubes were pre-incubated at RT for 5 min and then placed back on ice. After the addition of templates, reaction mixtures were incubated at 37°C for 1 h. Then, they were subjected to the PureLink RNase A treatment (20 mg/mL, Invitrogen, USA) for 15 min at RT. RNase A was preliminarily diluted 1:100 in PBS and 3 μL of this solution (600 ng of RNase A) was added to each reaction. Afterward, the samples were diluted in Laemmli sample buffer, incubated for 3 min at 70°C, and analyzed by SDS-PAGE (12% gel). Precision Plus Protein Kaleidoscope Prestained Protein Standards (Bio-Rad Laboratories, USA) were used as a protein ladder. The gel was scanned using the Typhoon FLA 9500 Biomolecular Imager (GE Healthcare, USA). A laser of 473 nm (blue LD laser) was used for excitation, and a DBR1 filter of 530 ± 20 nm was used for emission. Images were processed and visualized using the Image Lab software (version 6.0.1) (Bio-Rad Laboratories, USA). Levels of ErmC synthesis were normalized to a control sample (nuclease-free water) and visualized using the GraphPad Prism software (version 10.1.2).

### Western blot analysis

Following SDS-PAGE, proteins were transferred from the gel to a PVDF membrane (EcoTech Biotechnology, Turkey) using Trans-Blot SD Semi-Dry Transfer Cell (Bio-Rad Laboratories, USA) in Transfer Buffer (25 mM Tris base, 190 mM glycine, 20% [vol/vol] ethanol, pH 8.3). The membrane was blocked overnight at 4°C in 5% (wt/vol) BSA (Proliant Biologicals, USA) dissolved in TBST (20 mM Tris-HCl, 150 mM NaCl, 0.1% [vol/vol] Tween-20, pH 7.6) and then incubated overnight at 4°C with primary Monoclonal ANTI-FLAG M2 antibodies produced in mouse (Sigma-Aldrich, USA) diluted 1:1,000 in 5% BSA in TBST. After five 5-min washes in TBST, the membrane was incubated for 1.5 h at 4°C with secondary Goat Anti-Mouse IgG (H + L)-HRP-Conjugated antibodies (Bio-Rad Laboratories, USA) diluted 1:3,000 in 5% BSA in TBST. Then, the membrane was again washed five times in TBST, and the bands were developed using the WesternBright ECL kit (Advansta, USA) according to the manufacturer’s protocol. The membrane was visualized using the ChemiDoc Imaging System (Bio-Rad Laboratories, USA), and the images were analyzed using the Image Lab software (version 6.0.1) (Bio-Rad Laboratories, USA).

### Toeprinting assay

The toeprinting analysis was performed essentially as described in (71) with some modifications indicated below. Linear DNA templates *ermCL*, *ermBL*, and *ermDL* (Table S5) were generated by PCR using the following pairs of partially complementary primers—ErmCL-fwd + ErmCL-rev, ErmBL-fwd + ErmBL-rev, and ErmDL-fwd + ErmDL-rev, respectively (Table S4). The resulting templates (0.3 pmol) were expressed in a cell-free bacterial transcription-translation coupled system using the PURExpress *In Vitro* Protein Synthesis Kit (New England BioLabs, USA) in a total volume of 5 μL, according to the manufacturer’s guidelines. Antibiotics were added to the following final concentrations: thiostrepton (THS, 50 μM), borrelidin (BOR, 50 μM), azithromycin (AZI, 30 μM), azithromycin derivative with the linker (AZIL, 30 μM), and conjugates of azithromycin with benzoxaborole (AZI-BBs, 30 μM). “No drug” samples represent the addition of a 1% DMSO solution instead of antibiotics. Assembled reactions lacking DNA template were pre-incubated at RT for 5 min. Reactions were initiated by the addition of a DNA template and incubated at 37°C for 15 min. Then, 1 pmol of the ^32^P-labeled NV1 primer (Table S4) and 2 U of the AMV Reverse Transcriptase (Roche, Switzerland) were added, and the reaction tubes were additionally incubated at 37°C for 15 min. Reactions were terminated by the addition of 1 μL of 10 M NaOH, followed by incubation at 37°C for 15 min. The pH was further neutralized by the addition of an equivalent amount of concentrated HCl and 200 μL of Resuspension Buffer (0.3 M CH_3_COONa [pH 5.5], 5 mM EDTA, 0.5% [wt/vol] SDS). Thereafter, cDNA fragments were purified using the QIAquick PCR Purification Kit (Qiagen, Germany) according to the manufacturer’s protocol. Resulting cDNA solutions were dried at 95°C and dissolved in 10 μL of Formamide Loading Buffer (98% [vol/vol] formamide, 10 mM EDTA [pH 8.0], 0.1% [wt/vol], bromophenol blue, 0.1% [wt/vol] xylene cyanol). Sequencing reactions were performed using the USB Thermo Sequenase Cycle Sequencing Kit (Affymetrix, USA) according to the manufacturer’s protocol. Toeprinting samples, along with sequencing reactions, were resolved on a 6% polyacrylamide sequencing gel containing 7 M urea in TBE buffer (90 mM Tris base, 90 mM boric acid, 2 mM EDTA, pH 8.3). After electrophoresis, the gel was transferred onto Whatman cellulose chromatography paper (3MM, 46 × 57 cm) (GE Healthcare, USA), dried using the Savant Universal Vacuum System Plus with VaporNet (UVS400A, Thermo Fisher Scientific, USA) at 80°C for 1 h, and exposed to the phosphorimager screen overnight. The screen was scanned using the Typhoon FLA 9500 Biomolecular Imager (GE Healthcare, USA), and the images were processed using the Image Lab software (version 6.0.1) (Bio-Rad Laboratories, USA).

### Toe-seq analysis

#### Preparation of samples for Toe-seq

The Toe-seq analysis of AZI and AZI-BB2 context specificity was performed as reported in (47, 72). In brief, a library of linear DNA templates was generated by PCR amplification. One template consisted of a T7 promoter, a 5′ UTR (one of three types), an ORF containing a start codon, 30 randomized nucleotides, seven constant codons, and a stop codon, followed by a 3′ UTR (one of two types), a molecular barcode represented by 15 randomized nucleotides, and an annealing region of the RT primer (Table S4). The library was sequenced using NGS to obtain a dictionary representing each molecular barcode matched up with its ORF sequence and both UTR sequences. Then the library of DNA templates was expressed in a cell-free bacterial transcription-translation coupled system using the PURExpress *In Vitro* Protein Synthesis Kit (New England BioLabs, USA) in the presence of 50 μM AZI or AZI-BB2. Nuclease-free water served as a control. All reactions were prepared in two replicates. Reverse transcription using the AMV Reverse Transcriptase (Roche, Switzerland) and the Cy5-labeled RT primer (Table S4) was further applied to synthesize cDNA fragments. In general terms, the length distribution of the resulting cDNA fragments would indicate the position of *E. coli* ribosomes stalled during translation, whereas the molecular barcode would allow revealing the nucleotide context of the stall sites. Purified cDNA fragments were further elongated at their 3′ end by Terminal Transferase (New England BioLabs, USA) supplied with dATP. Poly(A)-tailed cDNA was then converted into dsDNA using the Klenow Fragment (3′→5′ exo-) (New England BioLabs, USA) and additionally amplified by PCR. Purified PCR products were subjected to NGS library preparation using the MGIEasy Universal DNA Library Prep Set (MGI Tech, China) and sequenced on the MGIseq-2000 (MGI Tech, China) at the Genomics Core Facility (ICBFM SB RAS, Russia).

#### Computational processing of Toe-seq data

Raw FastQ sequencing data were processed according to the procedure reported in a previous study (47). All the computations described below were performed using in-house Bash and Python scripts exactly as described in (72). Parameters of the resulting Toe-seq data sets are given in the Table S6. For each mRNA in a data set, read counts per nucleotide position were calculated as the number of reads ending at each position. Reads terminated at 5′ UTR were summed and denoted as FL values representing the quantity of full-length cDNA fragments. Thereafter, read counts per position were normalized to the total number of mapped reads and then multiplied by 10^6^, resulting in the so-called counts per million (CPM). For each sense codon, normalized read counts associated with positions +16, +17, and +18 relative to the first nucleotide of the codon were summed up and divided by the total number of CPM of a given mRNA (*CPM_mRNA_*). The resulting values reflect ribosome density distribution along the coding sequence and correspond to codons located in the P site of the ribosome. In addition, all CPM values associated with positions located between the start codon and the start codon +15 nucleotides were summed with the previously calculated FL values and divided by the corresponding *CPM_mRNA_*. Next, we normalized the data of antibiotic-treated samples to that of control samples (nuclease-free water) by subtracting the corresponding ribosome densities. This step additionally allows us to exclude non-specific reverse transcriptase products from further consideration. The resulting positive values represent the distribution of ribosome stalling probability (*StopProbability*) along the ORF sequence, whereas the negative values should be interpreted as truly “impossible” sites for ribosome stalling, not merely “zero probability”. The serial number of the codon associated with the maximum probability of ribosome stalling (*MaxStopProbability*) was defined as the stop position (*StopPosition*) at a given mRNA. In the case of mRNAs containing a premature stop codon, only appropriate *StopProbability* values were considered to determine *MaxStopProbability* and *StopPosition*. All *StopProbability* values that fall outside the real range of ORF were excluded from the analysis in advance. Since the accuracy of the *MaxStopProbability* score depends on the mRNA coverage (denoted here as *CPM_mRNA_*), we introduced a *Likelihood* metric that takes into account the reliability of the calculated *MaxStopProbability* over the entire data set. This metric was defined as follows:


$$Likelihood_{i}=MaxStopProbability_{i}\cdot 2^{lg\left(\frac{CPM_{mRNA,i}\cdot CPM_{mRNAc,i}}{\sum_{j}CPM_{mRNA,j}\cdot CPM_{mRNAc,j}}\right)}$$

where ${CPM}_{mRNA,i}$ and ${CPM}_{mRNAc,i}$ stand for the total number of CPM attributed to a given mRNA (with the barcode = i) and obtained from antibiotic-treated and control samples, respectively. It is worth noting that we use *Likelihood* as a relative rather than an absolute metric. Likewise, we introduced another parameter named *cpmRNAratio*, which represents the similarity in mRNA coverage between two samples and is calculated as follows:


$$cpmRNAratio_{i}=\;\frac{CPM_{mRNA,i}}{CPM_{mRNAc,i}}$$

The following filter criteria were further applied to narrow down the most relevant set of mRNA from a data set: (i) *MaxStopProbability* score should be higher than 0, (ii) *cpmRNAratio* should be within the interquartile range (IQR), (iii) mRNAs with *CPM_ORF_*, *CPM_mRNAc_*, and *Likelihood* values below the corresponding 25^th^ percentile should be filtered out. Here, *CPM_ORF_* is the number of CPM in the mRNA coding region. The resulting data sets were used to calculate the distribution of ribosome stalling sites along ORF, as well as to reveal codons and motifs associated with antibiotic-induced ribosome stalling. In order to consider predominantly those events that correspond to the highest efficiency of ribosome stalling, we used the *MaxStopProbability* adjustment when calculating the frequencies of the events. All processed Toe-seq data and computation results are provided in the accompanying Excel Tables (Tables S7 to S15). The obtained data were visualized using the GraphPad Prism software (version 10.1.2).

### X-ray crystallographic structure determination

Wild-type 70S ribosomes from *T. thermophilus* (strain HB8) were prepared as described previously (73–76). Synthetic mRNA with the sequence 5′-GGC-AAG-GAG-GUA-AAA-AUG-UUC-UAA-3′ containing Shine-Dalgarno sequence (underlined), followed by methionine (AUG) and phenylalanine (UUC) codons was obtained from Integrated DNA Technologies (USA). Non-hydrolyzable aminoacylated Phe-tRNA^Phe^ and fMet-tRNA_i_^Met^ were prepared as described previously (7, 75, 77–79).

AZI-BB2 compound was co-crystallized with the 70S ribosome in a functional state corresponding to the PTC pre-peptide bond formation configuration with unreacted aminoacylated Phe-tRNA^Phe^ and fMet-tRNA_i_^Met^ in the A and P sites, respectively. Complexes of the wild-type *T. thermophilus* tightly-coupled 70S ribosomes with mRNA, aminoacylated A-site Phe-tRNA^Phe^, and aminoacylated P-site fMet-tRNA_i_^Met^ were formed by programming 5 μM 70S ribosomes with 10 μM mRNA and 20 μM P- and A-site tRNAs. The complex was formed in the buffer containing 5 mM HEPES-KOH (pH 7.6), 50 mM KCl, 10 mM NH_4_Cl, and 10 mM Mg(CH_3_COO)_2_, and then crystallized in the buffer containing 100 mM Tris-HCl (pH 7.6), 2.9% (vol/vol) PEG-20K, 9%–10% (vol/vol) MPD, 175 mM arginine, and 0.5 mM β-mercaptoethanol. Crystals were grown by the vapor diffusion method in sitting drops at 19°C, stabilized, and cryo-protected stepwise using a series of buffers with increasing MPD concentrations (25%, 30%, and 35%) until reaching the final concentration of 40% (vol/vol) MPD as described previously (7, 75–78,79,80). AZI-BB2 compound was included in the crystallization mixture (250 µM) and also added to the final 40% MPD stabilization buffers (250 µM). After stabilization and cryo-protection, crystals were flash-frozen using a nitrogen cryo-stream at 80 K (Oxford Cryosystems, UK).

Collection and processing of the X-ray diffraction data, model building, and structure refinement were performed as described in our recent reports (7, 54, 77–78,79,80). Diffraction data were collected at beamlines 24ID-C and 24ID-E at the Advanced Photon Source (Argonne National Laboratory, USA). A complete data set for each complex was collected using 0.979 Å irradiation at 100 K from multiple regions of the same crystal, using 0.3-degree oscillations. Raw data were integrated and scaled using XDS software (version from Jan 10, 2022) (81). Molecular replacement was performed using PHASER from the CCP4 program suite (version 7.0) (82). The search model was generated from the previously published structures of *T. thermophilus* 70S ribosome with bound mRNA and aminoacylated tRNAs (PDB entry 6XHW (7)). Initial molecular replacement solutions were refined by rigid-body refinement with the ribosome split into multiple domains, followed by positional and individual B-factor refinement using PHENIX software (version 1.17) (83). Non-crystallographic symmetry restraints were applied to four parts of the 30S ribosomal subunit (head, body, spur, and helix 44) and four parts of the 50S subunit (body, L1-stalk, L10-stalk, and C-terminus of the L9 protein). Structural models were built in Coot (version 0.8.2) (84). Structural models and restraints for AZI-BB2 were generated using PRODRG software from the CCP4 suite (85). The statistics of data collection and refinement are compiled in Table S3. All figures showing atomic models were rendered using PyMol software (www.pymol.org).

## Data Availability

Coordinates and structure factors were deposited in the RCSB Protein Data Bank with accession code 10PX for the wild-type *T. thermophilus* 70S ribosome in complex with benzoxaborole derivative of azithromycin (AZI-BB2), mRNA, aminoacylated A-site Phe-tRNA^Phe^, aminoacylated P-site fMet-tRNA_i_^Met^, and deacylated E-site tRNA^Phe^. The Toe-seq sequencing data have been deposited to the NCBI BioProject database under accession number PRJNA1304769. Bioinformatics scripts for Toe-seq analysis are available at https://github.com/kabilov/Publication_scripts/tree/main/2024_Toe-seq. Other relevant data that support this study are available in the main text, supplementary information, or from the corresponding authors upon reasonable request.

## SUPPLEMENTAL MATERIAL

Supplemental Material aac.01539-25-s0001.pdf Fig. S1 to S15 and Tables S1 to S6.

Tables S11 to S15 aac.01539-25-s0002.xlsx Toe-seq analysis of ribosome stall sites and frequencies of stall motifs.

Table S7 aac.01539-25-s0003.xlsx Processed Toe-seq data for AZI-treated sample (replicate 1).

Table S8 aac.01539-25-s0004.xlsx Processed Toe-seq data for AZI-treated sample (replicate 2).

Table S9 aac.01539-25-s0005.xlsx Processed Toe-seq data for AZI-BB2-treated sample (replicate 1).

Table S10 aac.01539-25-s0006.xlsx Processed Toe-seq data for AZI-BB2-treated sample (replicate 2).

ASM does not own the copyrights to Supplemental Material that may be linked to, or accessed through, an article. The authors have granted ASM a non-exclusive, world-wide license to publish the Supplemental Material files. Please contact the corresponding author directly for reuse.
