# Supplementary material for: Benzoxaborole-modified azithromycins inhibit translation without inducing ermC expression
Source: Antimicrob Agents Chemother. 2026 Mar 24;70(5):e01539-25. doi: 10.1128/aac.01539-25 (PMC13148051; doi:10.1128/aac.01539-25)
Supplement: Supplemental Material — Fig. S1 to S15 and Tables S1 to S6. [file aac.01539-25-s0001.pdf]

Supplementary Materials

Benzoxaborole-modified azithromycins inhibit translation without inducing *ermC* expression

Inna A. Volynkina[1,2,*], Michael O. Bortyazh[1,3,*], Chih-Wei Chen[4,*], Andrey G. Tereshchenkov[3], Anastasiia O. Karakchieva[1], Dmitrii A. Lukianov[1,2], Ekaterina S. Komarova[1,3], Alexey E. Tupikin[5], Dmitry A. Skvortsov[1], Anna N. Tevyashova[6], Alexander S. Tikhomirov[7], Vadim N. Tashlitsky[1], Marsel R. Kabilov[5], Andrey E. Shchekotikhin[8], Olga A. Dontsova[1,2,3,9], Yury S. Polikanov[4,10,11], and Petr V. Sergiev[1,2,3]

[1] Department of Chemistry, Lomonosov Moscow State University, Moscow, Russia

[2] Center for Molecular and Cellular Biology, Moscow, Russia

[3] A.N. Belozersky Institute of Physico-Chemical Biology, Lomonosov Moscow State University, Moscow, Russia

[4] Department of Biological Sciences, University of Illinois at Chicago, Chicago, Illinois, USA

[5] Institute of Chemical Biology and Fundamental Medicine, Siberian Branch of the Russian Academy of Sciences, Novosibirsk, Novosibirsk Region, Russia

[6] School of Science, Constructor University, Bremen, Germany

[7] Laboratory of Synthesis of Antibiotics Overcoming Drug Resistance, Gause Institute of New Antibiotics, Moscow, Russia

[8] Laboratory of Chemical Transformation of Antibiotics, Gause Institute of New Antibiotics, Moscow, Russia

[9] Department of Functioning of Living Systems, Shemyakin-Ovchinnikov Institute of Bioorganic Chemistry, Moscow, Russia

[10] Department of Pharmaceutical Sciences, University of Illinois at Chicago, Chicago, Illinois, USA

[11] Center for Biomolecular Sciences, University of Illinois at Chicago, Chicago, Illinois, USA

* Authors equally contributed to this work.

SUPPLEMENTARY FIGURES

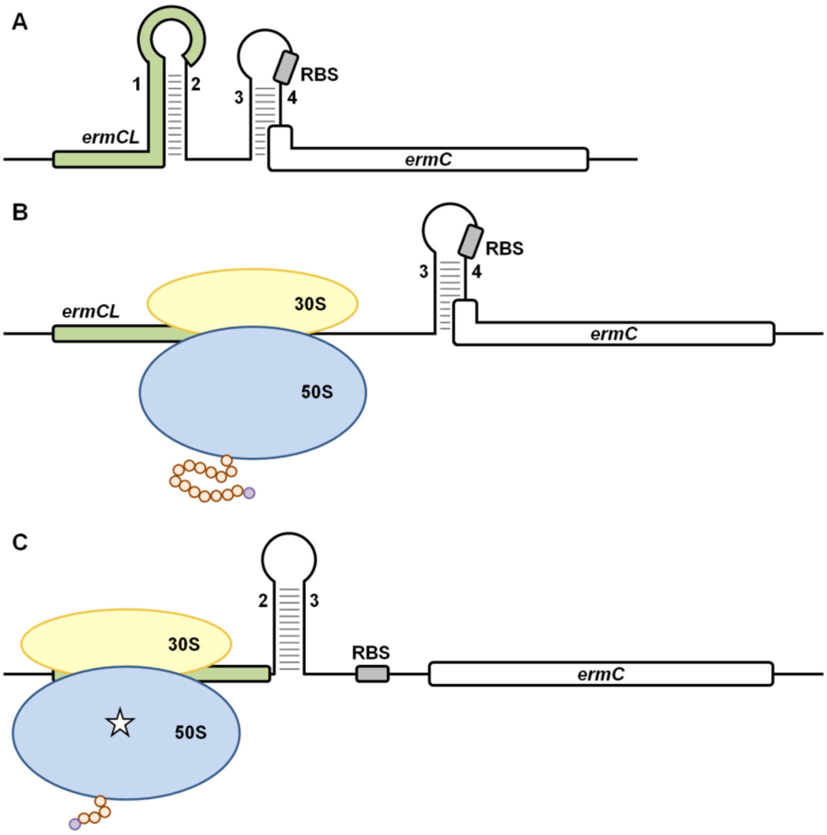

Figure S1. Regulation of *ermC* expression. (**A**) When not being translated, *ermC* mRNA forms two hairpins. The hairpin 1:2 is constituted by complementary segments 1 and 2 and harbors a 3′ region of the *ermCL* leader open reading frame. The hairpin 3:4 consists of complementary segments 3 and 4 and hides the first two *ermC* codons, as well as the ribosome binding site (RBS), which prevents downstream translation. (**B**) Regular translation of *ermCL* results in melting the hairpin 1:2, but does not interfere with the hairpin 3:4. When the ribosome is located at the stop codon, it covers the segment 2, thus impeding the formation of the hairpin 2:3. (**C**) Macrolide antibiotics (shown with an asterisk) arrest the ribosome at a certain position of *ermCL*, which is enough to coat the segment 1, but not yet the segment 2. This allows the mRNA to fold into a more energetically advantageous hairpin 2:3, which makes the RBS element available for ribosome binding and favors *ermC* translation.

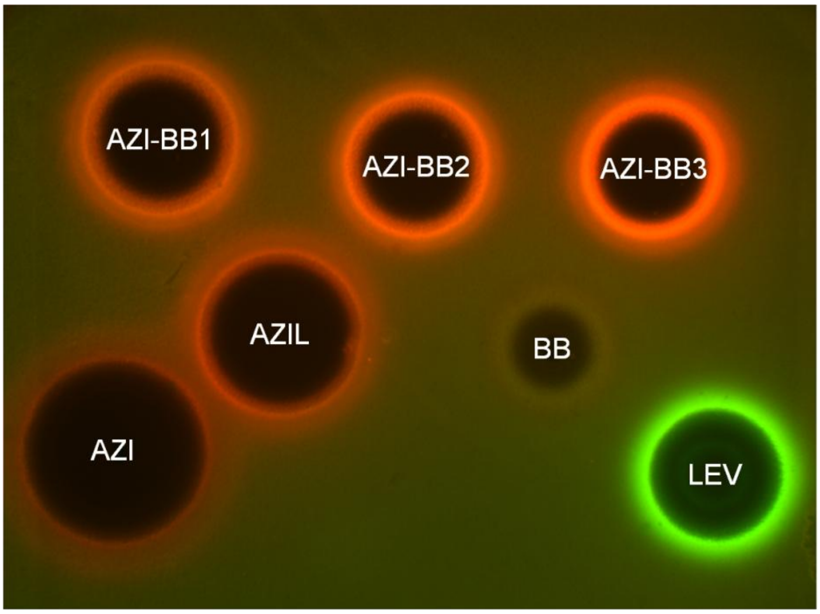

Figure S2. Reporter induction demonstrated by AZI-BB conjugates using the pDualrep2 double reporter system. An agar plate was coated with the *E. coli ΔtolC* pDualrep2 strain and spotted with azithromycin (AZI), its derivatives (AZIL, AZI-BB1-3), the benzoxaborole moiety (BB) and levofloxacin (LEV). The plate was scanned in “Cy3-blot” (for TurboRFP fluorescence) and “Cy5-blot” (for Katushka2S fluorescence) channels, shown as green and red pseudocolors, respectively. The pDualrep2 system consists of two reporter genes, *katushka2S* and *turboRFP* (1). The expression of the far-red fluorescent protein gene *katushka2S* in the zone of antibiotic sublethal concentrations occurs in response to ribosome stalling during translation of the modified *trpL2A* leader sequence. The expression of the red fluorescent protein gene *turboRFP* indicates the induction of the SOS response triggered by the accumulation of DNA damage.

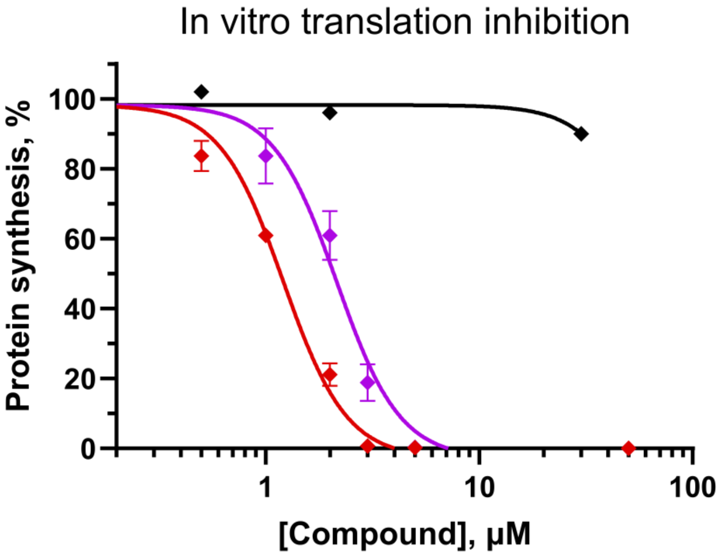

Compound	IC_{50}, µM
AZI	1.1 ± 0.1
AZIL	2.1 ± 0.2
BB	NA

Figure S3. Inhibition of protein synthesis *in vitro* by AZI, AZIL, and BB moiety in a bacterial cell-free system. Inhibition of protein synthesis by increasing concentrations of AZI, AZIL and BB was assessed *in vitro* in a cell-free bacterial translation system. Relative maximum Fluc accumulation rates are shown. Error bars represent standard deviation. All reactions were repeated at least two times. Calculated IC_{50} values and 95% confidence intervals are shown in the table. NA, not applicable.

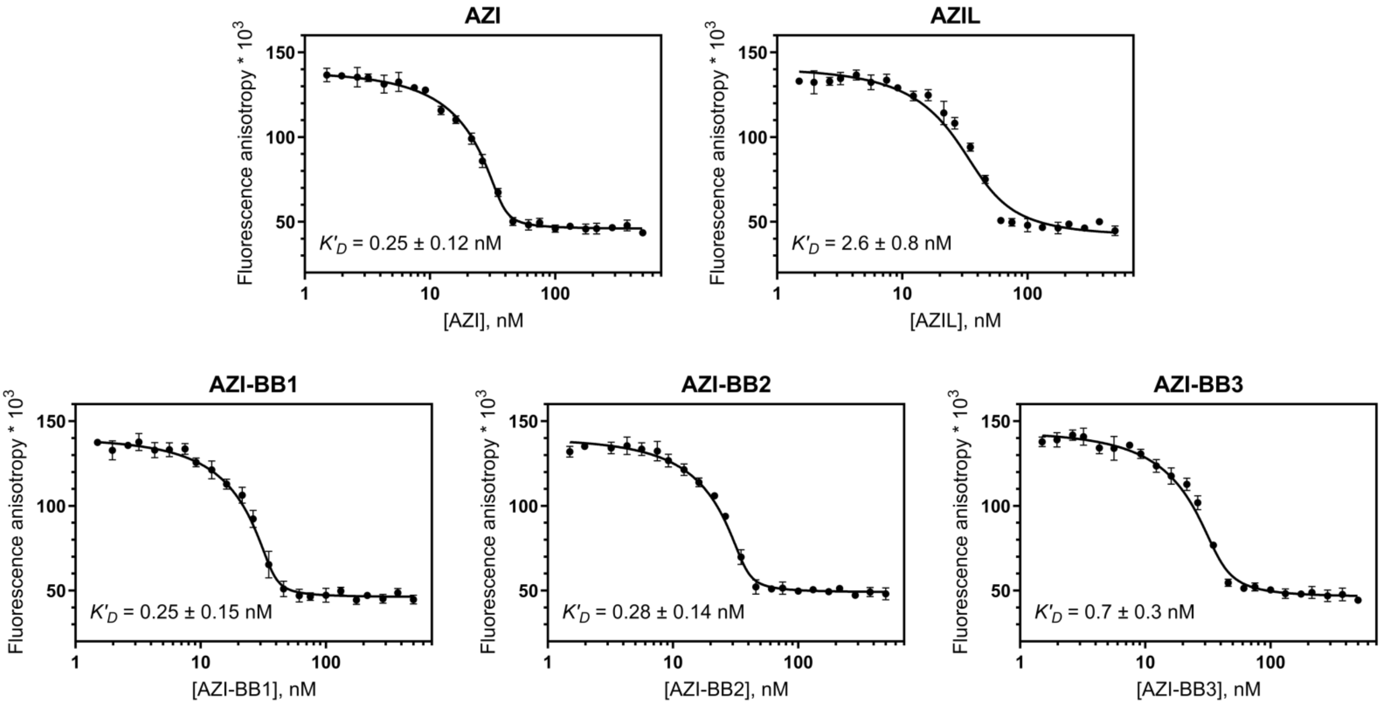

Figure S4. Displacement of BODIPY-ERY by AZI, AZIL, and AZI-BB conjugates from the *E. coli* 70S ribosomes. Competition-binding assay revealing a decrease in fluorescence anisotropy upon an increase in concentration of compounds. Error bars represent standard deviation. The resulting average apparent dissociation constants (K'_D) and their 95% confidence intervals are shown on each plot. All reactions were repeated at least four times.

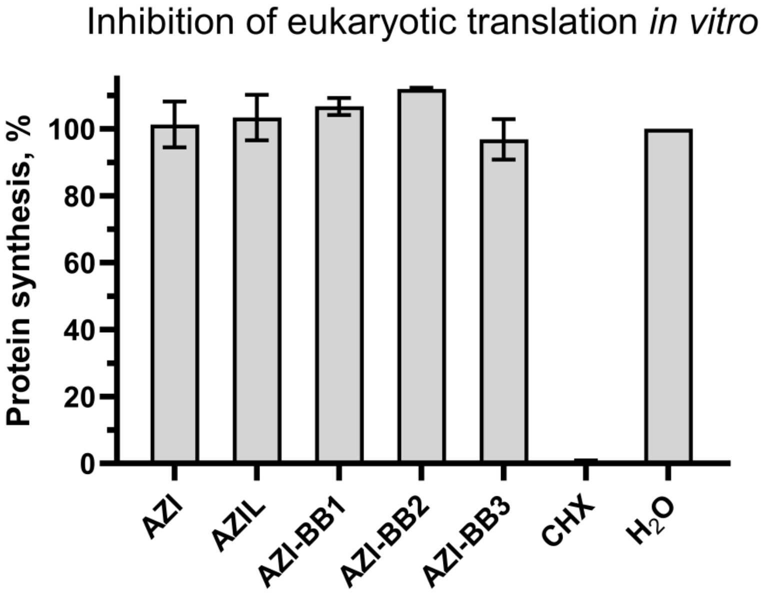

Figure S5. Inhibition of protein synthesis *in vitro* by AZI, AZIL, and AZI-BB conjugates in HEK293T whole-cell lysate system. Inhibition of protein synthesis was assessed *in vitro* in a mammalian cell-free translation system based on HEK293T whole-cell extracts. Relative maximum Fluc accumulation rates are shown. CHX, cycloheximide, was used as a reference agent. Error bars represent standard deviation. All compounds were tested at a final concentration of 50 μM. All reactions were repeated at least two times.

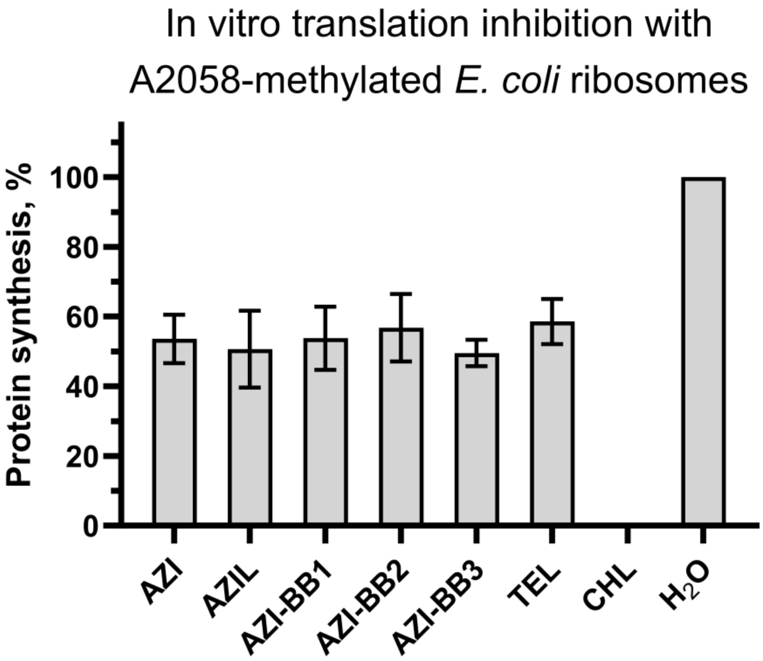

Figure S6. Inhibition of protein synthesis *in vitro* by AZI, AZIL, and AZI-BB conjugates in the presence of A2058-methylated 70S *E. coli* ribosomes. Inhibition of protein synthesis was assessed *in vitro* in a bacterial cell-free Δ ribosome translation system. The reactions were supplied with purified A2058-methylated *E. coli* ribosomes. The relative amount of Fluc accumulated during translation is shown. CHL, chloramphenicol, was used as a reference agent. Error bars represent standard deviation. All compounds were tested at a final concentration of 50 μM. All reactions were repeated at least three times.

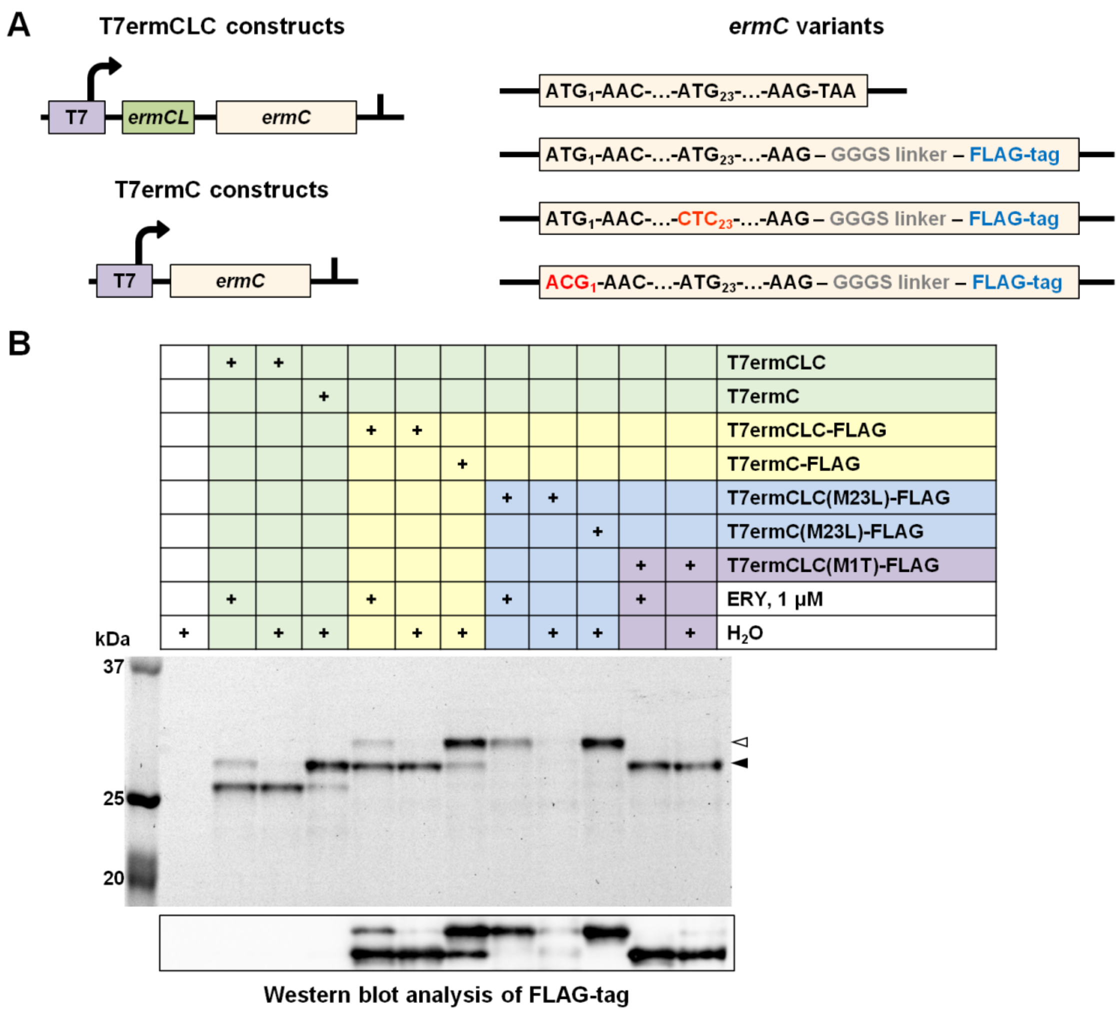

Figure S7. Induction of ErmC methyltransferase synthesis *in vitro* by treatment with subinhibitory concentration of ERY. (**A**) A schematic representation of the DNA constructs used in this work. Two sets of DNA constructs were prepared, with either the whole *ermCL-ermC* operon (T7ermCLC constructs) or just the *ermC* ORF (T7ermC constructs) under the control of the T7 promoter. In both cases, four variants of the *ermC* ORF were used, schematically depicted on the right. Constructs T7erm(CL)C-FLAG additionally encode a FLAG tag via the GGGS linker at the 3′ end of the *ermC* ORF. Moreover, the 23rd ATG codon was mutated to CTC (Leu), resulting in the T7erm(CL)C(M23L)-FLAG constructs essential to distinguish between the full-length ErmC and its shorter isoform. For the same purpose, the 1st ATG codon was also mutated to ACG (Thr), resulting in the T7ermCLC(M1T)-FLAG construct. (**B**) Gel electrophoresis analysis of BODIPY-Lys-labeled proteins accumulated in the bacterial cell-free transcription-translation coupled system programmed with different T7ermCLC and T7ermC constructs in the presence or absence of 1 µM erythromycin (ERY). Positions of the full-length ErmC-FLAG protein and the shorter ErmC-FLAG isoform are indicated by white and black arrowheads, respectively. Western blot analysis of the same gel using anti-FLAG antibodies is provided below.

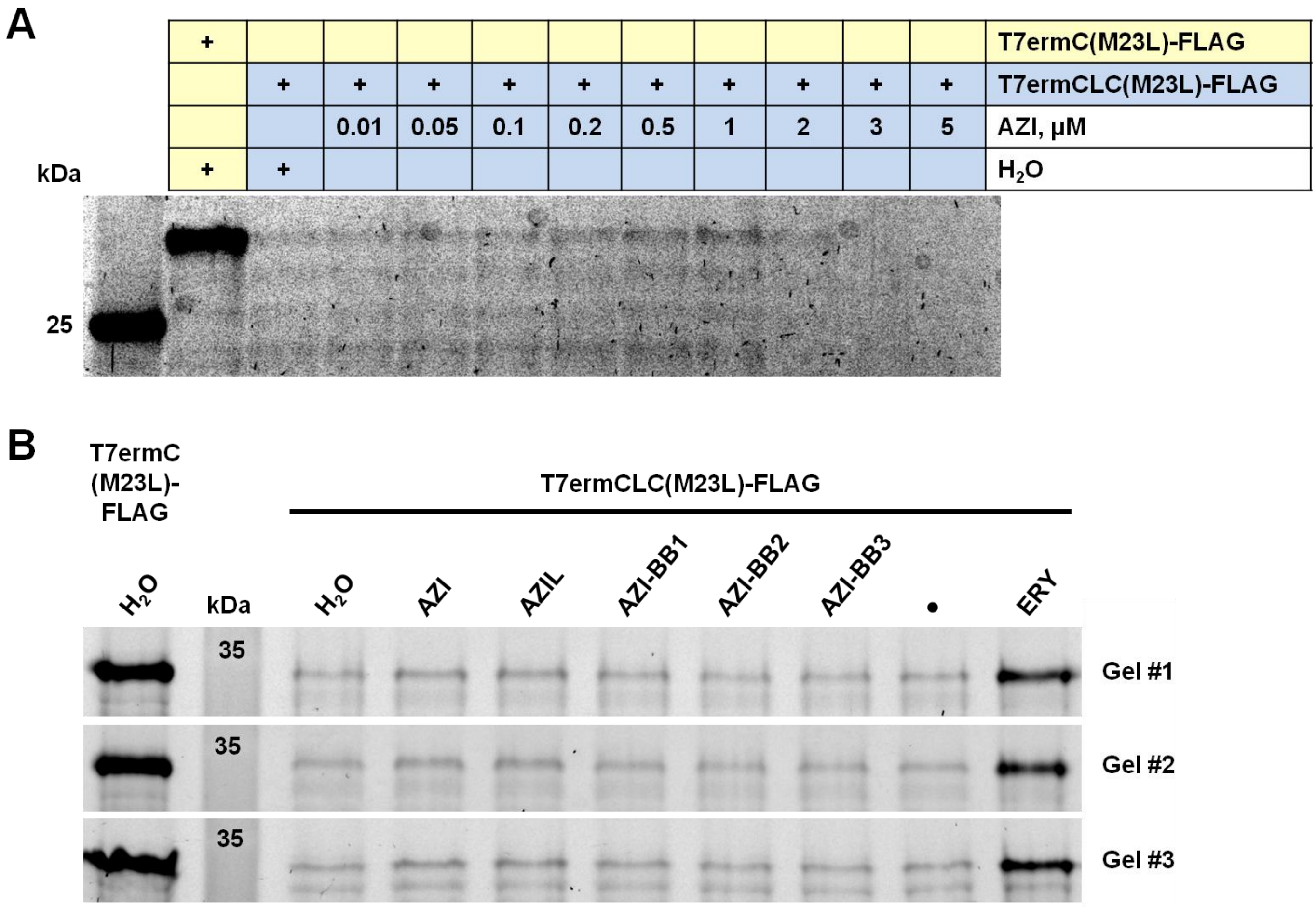

Figure S8. Induction of ErmC methyltransferase synthesis *in vitro* by treatment with AZI, AZIL, or AZI-BB conjugates. Gel electrophoresis analysis of BODIPY-Lys-labeled ErmC-FLAG accumulated in the bacterial cell-free transcription-translation coupled system programmed with either T7ermCLC(M23L)-FLAG or T7ermC(M23L)-FLAG DNA constructs. (**A**) Dependence of *ermC* expression on AZI concentrations. Induction of the full-length ErmC-FLAG synthesis is observed upon treatment with 0.05 – 2 µM AZI, with 1 µM concentration demonstrating the highest level of induction. (**B**) Comparison of AZI and its derivatives for the efficiency of induction of the full-length ErmC-FLAG synthesis. All compounds were tested at a final concentration of 1 µM. The intensity of gel bands was measured in three independent replicates and used to calculate levels of induction. The black dot corresponds to a compound which was not covered in this article.

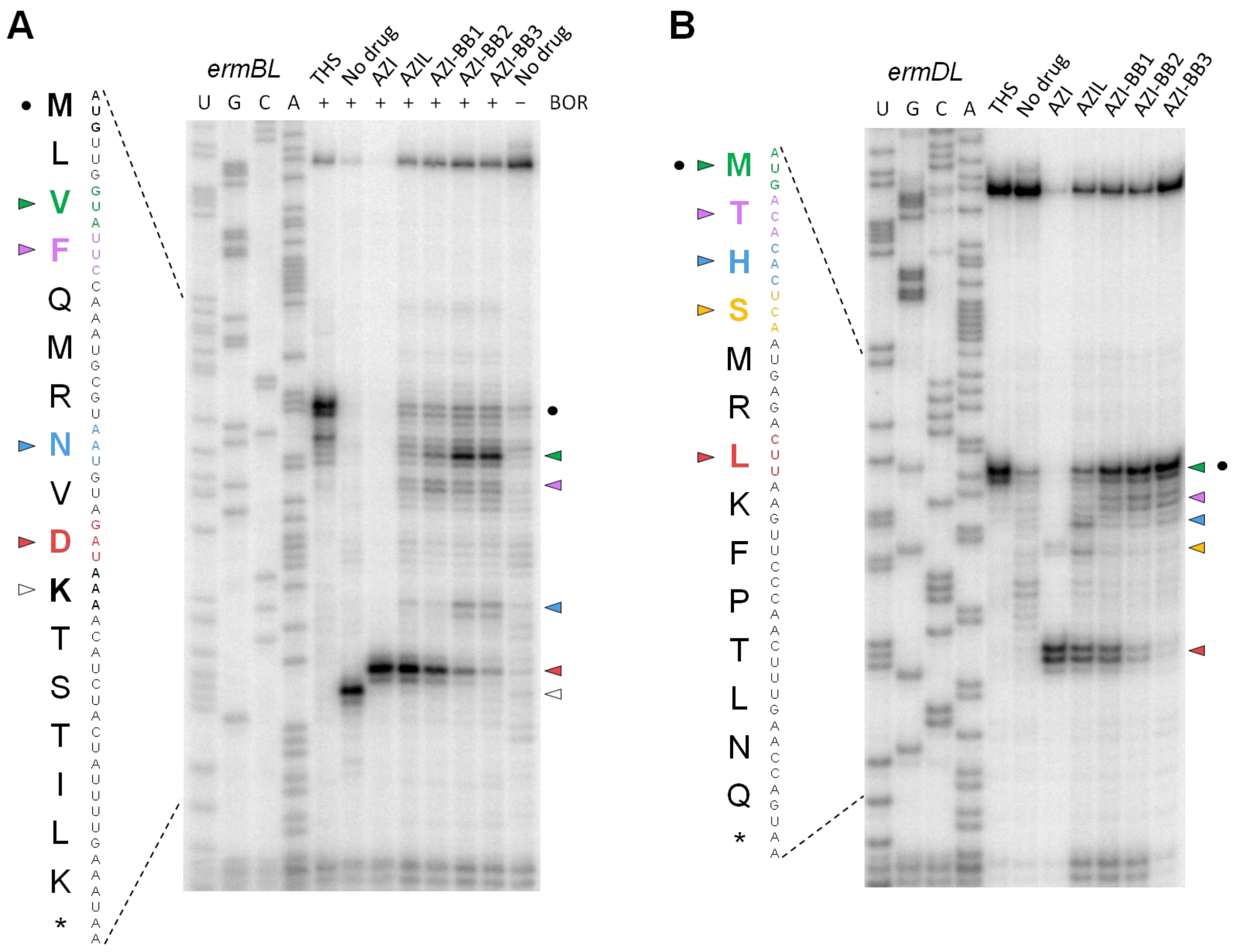

Figure S9. Toeprinting analysis of AZI-BB conjugates on *ermBL* and *ermDL* mRNAs. Toeprinting analysis of AZI derivatives on (**A**) *ermBL* and (**B**) *ermDL* mRNA templates in the presence (+) or absence (−) of borrelidin (BOR). Arrowheads indicate the toeprint bands corresponding to ribosomes stalled during translation. Codons located in the P site of stalled ribosomes are indicated with the same arrowheads in mRNA sequence. Ribosome stalling at the start codon (AUG) is marked by black dots. Thiostrepton (THS) was included to map the translation start site. AZI, AZIL, and AZI-BB1-3 were tested at a final concentration 30 μM. THS and BOR were used at 50 μM.

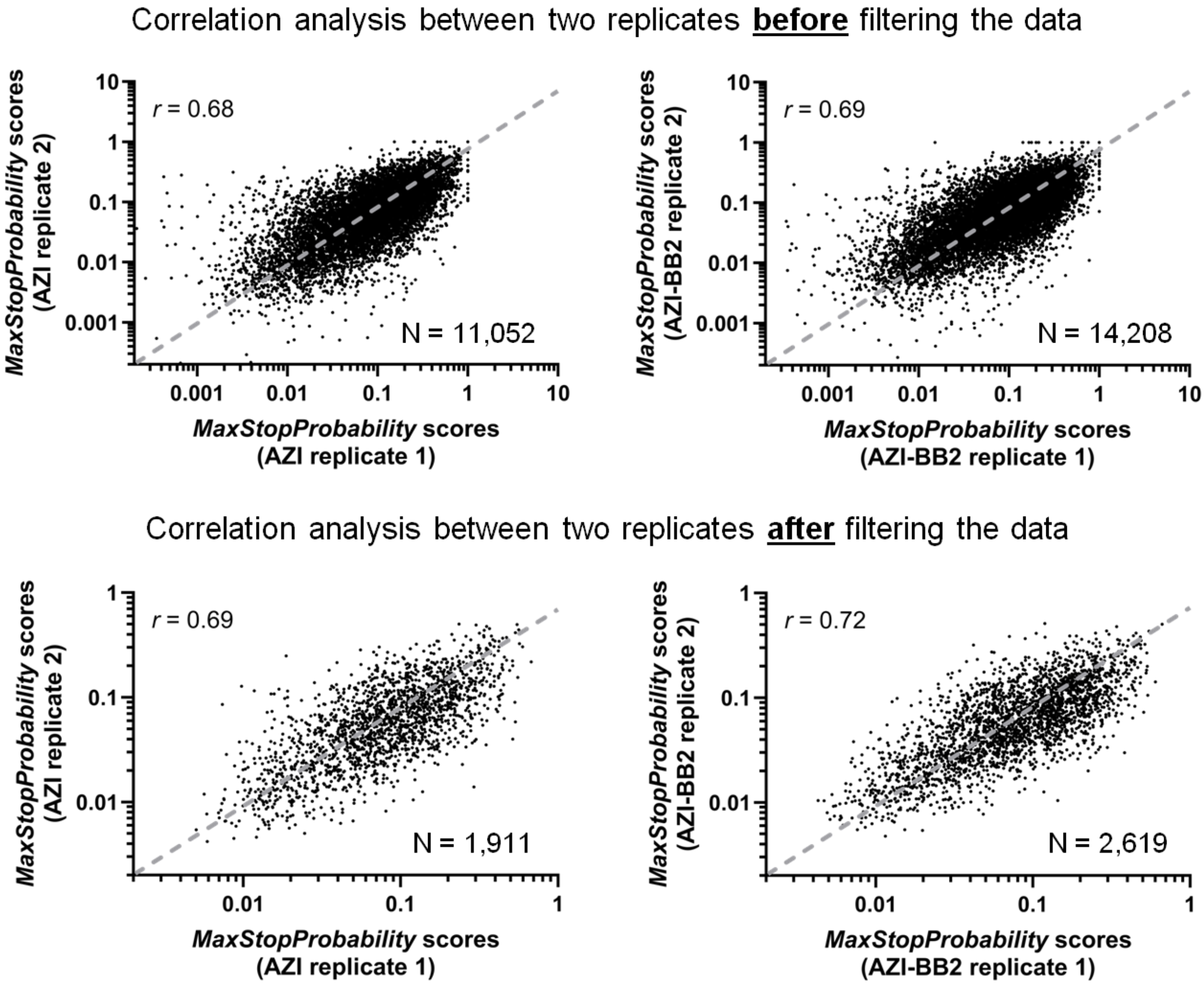

Figure S10. Correlation analysis of *MaxStopProbability* scores between two independent Toe-seq experiments. Toe-seq analysis of AZI and AZI-BB2 was performed in two biological replicates. For each NGS data set, *MaxStopProbability* scores were calculated to identify antibiotic-specific ribosome stalling sites. A subset of mRNAs associated with coincident stalling sites in both replicates was extracted for correlation analysis. In addition, only mRNAs with *MaxStopProbability* scores ≥ 0 were considered. The number of appropriate mRNAs (N) is presented on each plot. Spearman correlation coefficient (*r*) of the corresponding *MaxStopProbability* scores was calculated, p-value < 0.0001 (two-tailed). The trend line is shown as a gray dashed line.

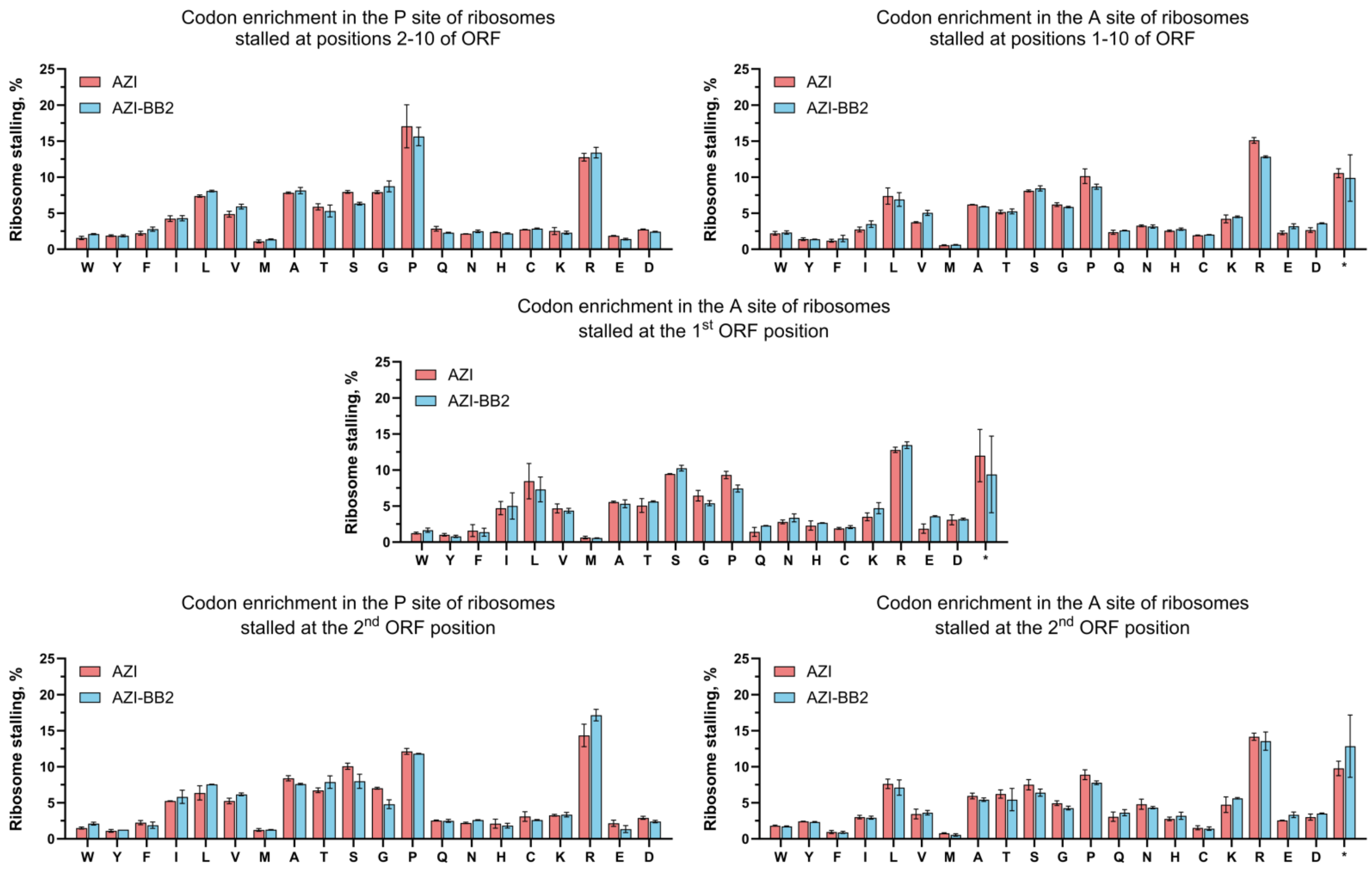

Figure S11. Enrichment of codons occupying the P and A sites of ribosomes stalled at the 1st and 2nd ORF positions in the presence of AZI or AZI-BB2. Enrichment is shown as the relative occurrence of specific amino acids encoded by the codons located in the P and A sites of arrested ribosomes. The calculated values were normalized using *MaxStopProbability* scores. An asterisk (*) denotes stop codons. Error bars indicate standard deviation.

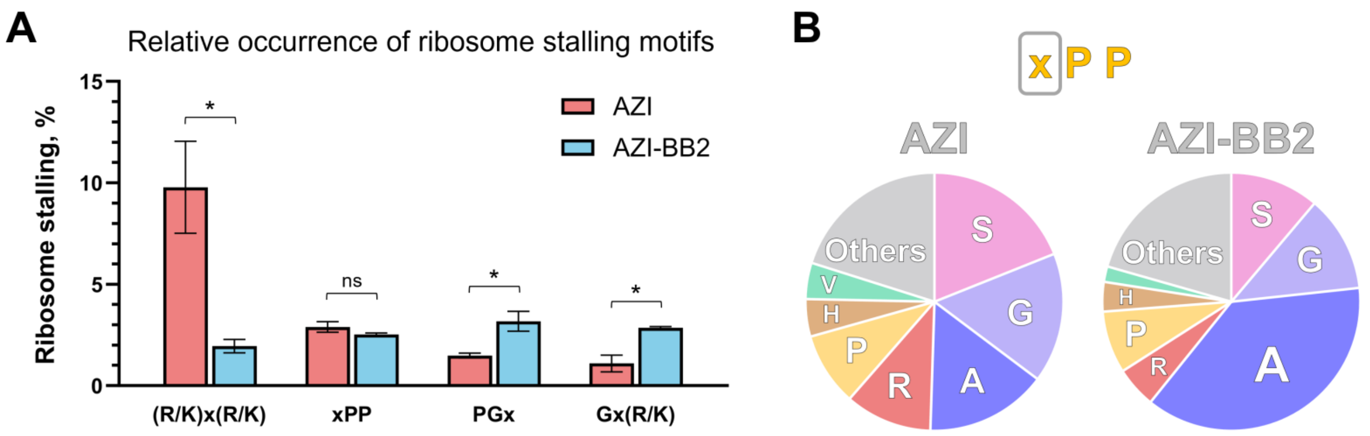

Figure S12. Relative occurrence of the selected motifs in AZI- and AZI-BB2-treated samples. (**A**) Relative occurrence of the selected stalling motifs representing the difference between AZI- and AZI-BB2-treated samples. The presented motif frequencies were adjusted for *MaxStopProbability* scores to account for the efficiency of ribosome stalling. p-value < 0.05 (*) based on one-tailed two sample (unpaired) t-test in n = 2 independent replicates. ns, not significant. Error bars show standard deviation. (**B**) Influence of the identity of amino acid fixed in the E site of the xPP stalling motif on the efficiency of ribosomal arrest induced by AZI or AZI-BB2. Pie-charts represent the mean values of two independent replicates, reflecting the relative occurrence of the most prevalent amino acids in the xPP motif.

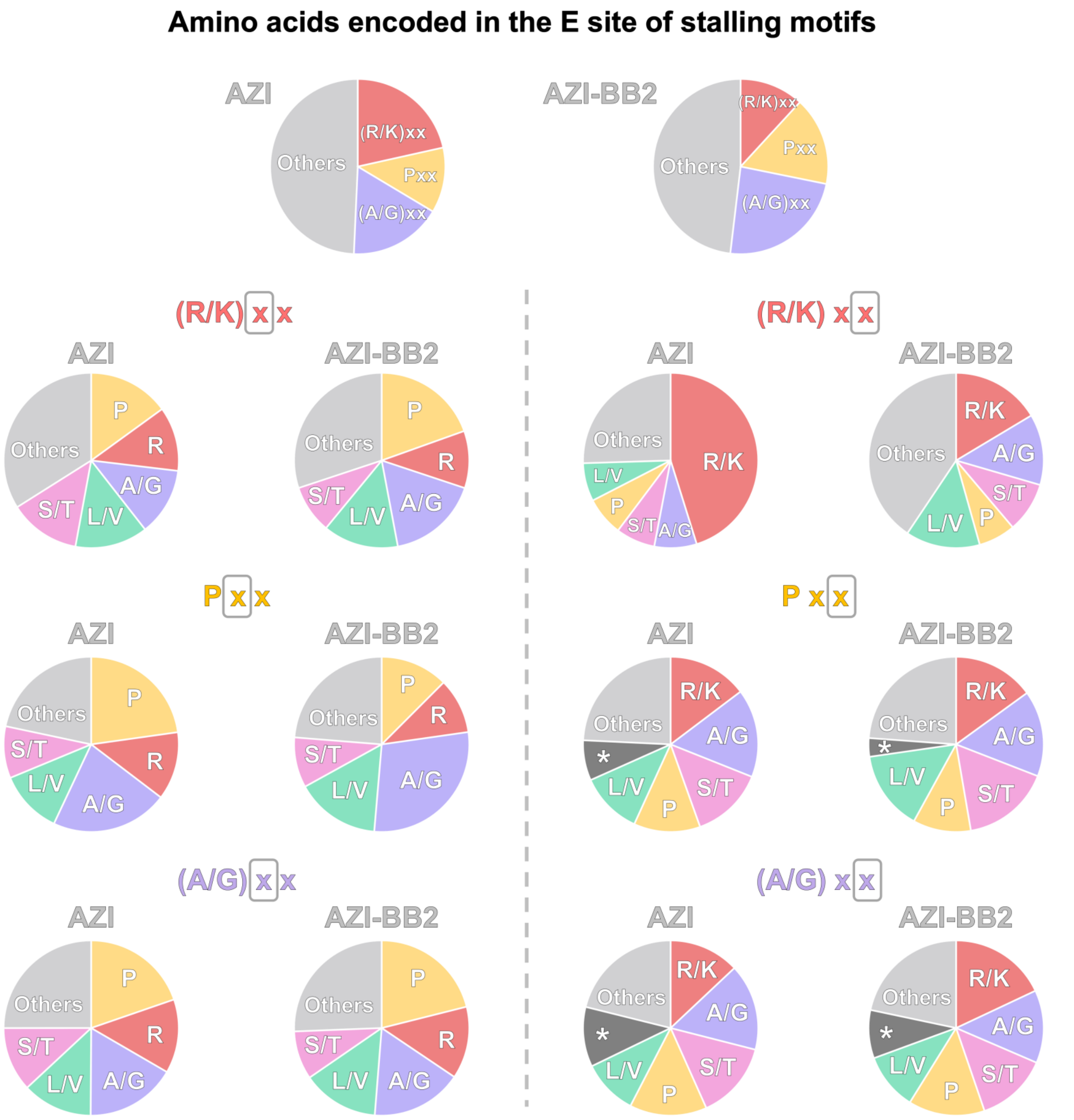

Figure S13. Relative occurrence of different prevalent motifs with fixed amino acids in the E site. Top pie-charts show the most frequent motifs associated with amino acids in the E site. The pie-charts below represent the relative occurrence of selected amino acids in the indicated subsets of stalling motifs. The pie-charts on the left side correspond to the variable P site. The pie-charts on the right side conform to the variable A site. An asterisk (*) denotes stop codons. All pie-charts represent the mean values of two independent replicates.

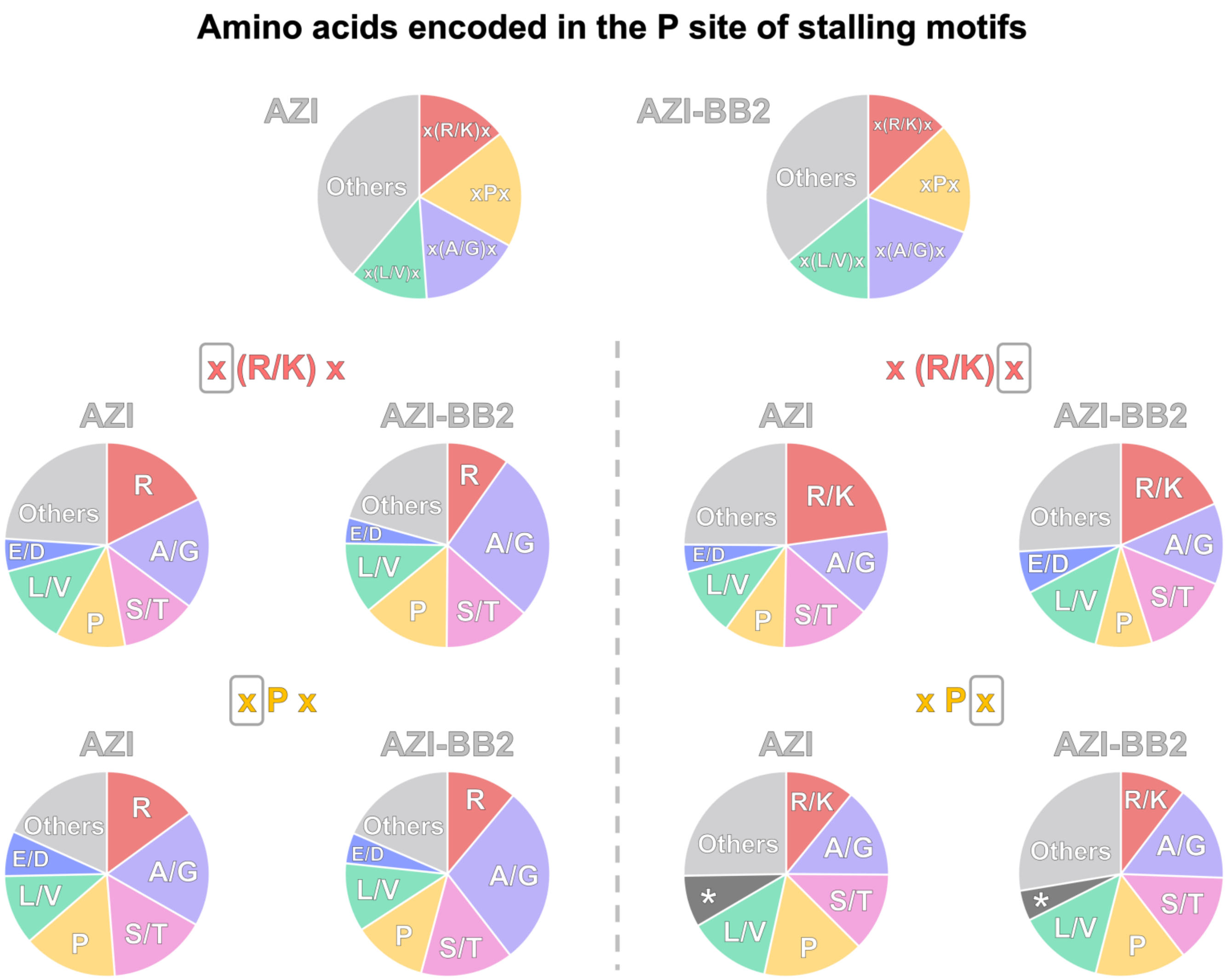

Figure S14. Relative occurrence of different prevalent motifs with fixed amino acids in the P site. Top pie-charts show the most frequent motifs associated with amino acids in the P site. The pie-charts below represent the relative occurrence of selected amino acids in the indicated subsets of stalling motifs. The pie-charts on the left side correspond to the variable E site. The pie-charts on the right side conform to the variable A site. An asterisk (*) denotes stop codons. All pie-charts represent the mean values of two independent replicates.

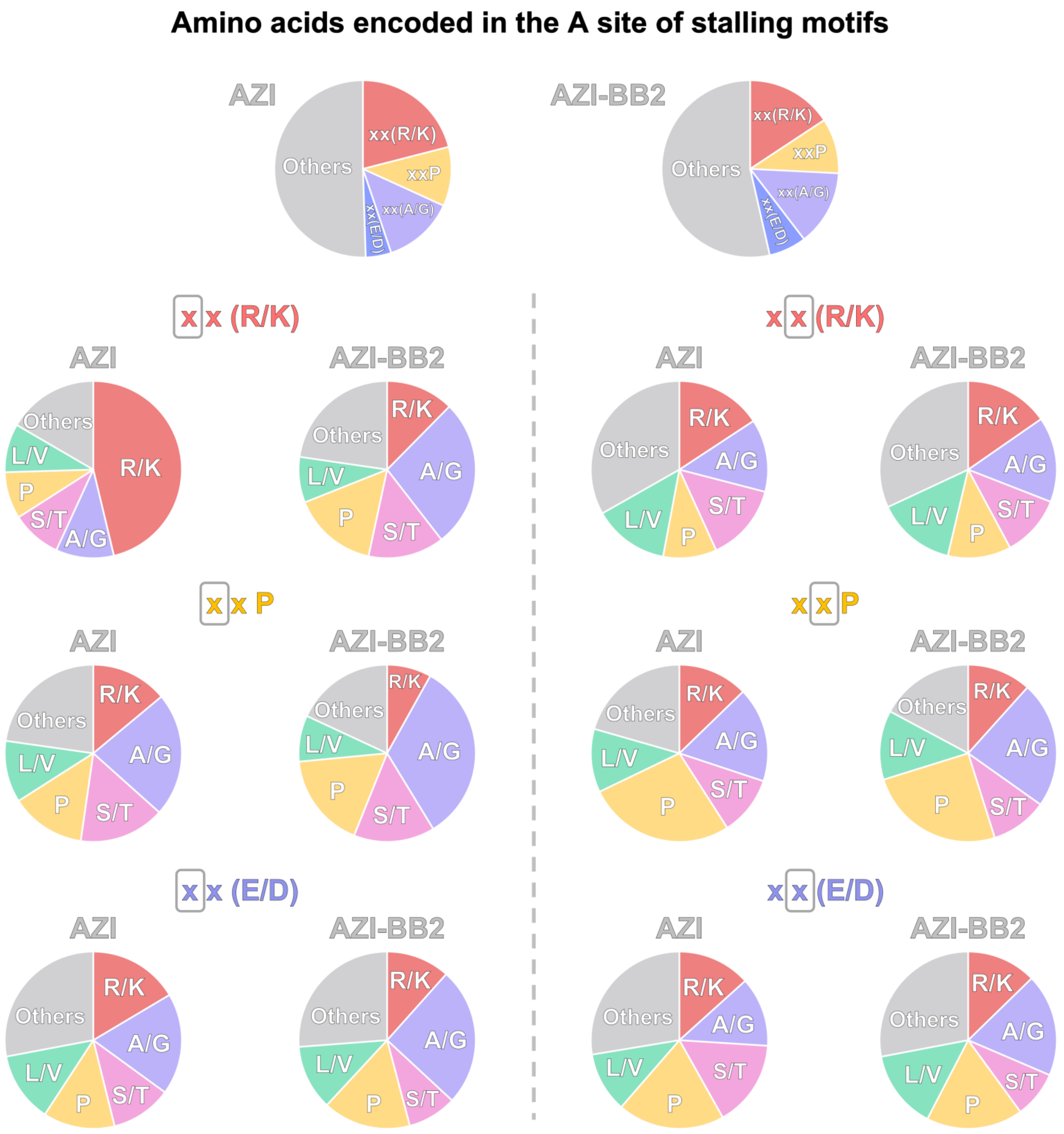

Figure S15. Relative occurrence of different prevalent motifs with fixed amino acids in the A site. Top pie-charts show the most frequent motifs associated with amino acids in the A site, and demonstrating the difference between AZI- and AZI-BB2-treated samples. The pie-charts below represent the relative occurrence of selected amino acids in the indicated subsets of stalling motifs. The pie-charts on the left side correspond to the variable E site. The pie-charts on the right side conform to the variable P site. All pie-charts represent the mean values of two independent replicates.

SUPPLEMENTARY TABLES

Table S1. Antibacterial activity of AZI-BB conjugates and reference compounds against *E. coli* strains with cMLS$_B$ phenotype.

	Compound, minimum inhibitory concentration (MIC), μM								
Strain	ERY	AZI	AZIL	AZI-BB1	AZI-BB2	AZI-BB3	BB	TEL	CHL
E. coli ΔtolC [a]	4	1	4	4	8	8	64	1	2
E. coli ΔtolC pErmC [b]	>128	>128	>128	64	64	64	64	8	2
E. coli SQ171 *ΔtolC* (WT) [c]	nt	1	2	4	4	4	nt	nt	1
E. coli SQ171 *ΔtolC* (A2058G) [d]	nt	>256	>256	128	128	256	nt	nt	2

[a] *E. coli ΔtolC*: macrolide-susceptible strain.

[b] *E. coli ΔtolC* pErmC: macrolide-resistant strain with cMLS$_B$ phenotype due to the constitutive expression of the *ermC* gene.

[c] *E. coli* SQ171 *ΔtolC* (WT): macrolide-susceptible strain.

[d] *E. coli* SQ171 *ΔtolC* (A2058G): macrolide-resistant strain with cMLS$_B$ phenotype due to the A2058G mutation in the 23S rRNA gene.

At least three replicates of MIC measurements were performed.

nt, not tested.

Table S2. Cytotoxic activity of AZI-BB conjugates against human cell lines of different etiology.

	Human cell lines, 50% cytotoxic concentration (CC_{50}), μM			
Compound	MCF7	VA13	A549	HEK293T
AZI	24.7 ± 2.4	18.6 ± 1.6	58.2 ± 5.1	23.6 ± 2.5
AZIL	>100	67.9 ± 9.8	>100	>100
AZI-BB1	5.7 ± 0.6	12.0 ± 0.4	28.8 ± 1.3	7.2 ± 0.6
AZI-BB2	15.0 ± 1.8	36.0 ± 1.8	63.4 ± 13.5	29.6 ± 1.9
AZI-BB3	>100	>100	>100	>100

At least three biologically independent replicates were performed.

Table S3. X-ray data collection and refinement statistics.

Crystal complex	***Ribosome***	**WT *Tth* 70S**
	A-site tRNA	**Phe-$tRNA^{Phe}$**
	P-site tRNA	**fMet-$tRNA_i^{Met}$**
	Bound drug	**AZI-BB2**
	PDB entry	**10PX**
**Data collection**		
Space group		$P2_12_12_1$
Cell dimensions		
a, b, c (Å)		208.22, 446.66, 615.32
α, β, γ (°)		90.0, 90.0, 90.0
Resolution (Å)		121-2.45 (2.51-2.45)[a]
R_{merge}		28.2 (254.6)
$I/\sigma I$		6.55 (0.85)[b]
Completeness (%)		99.9 (99.9)
Redundancy		8.49 (7.31)
**Refinement**		
Resolution (Å)		121-2.45
No. reflections		2,068,056
R_{work} / R_{free}		24.5/30.1
No. atoms		
Protein		90,996
Ligand/ion		204,653
Water		4,338
B factors		
Protein		53.3
Ligand/ion		51.2
Water		37.3
R.m.s. deviations		
Bond lengths (Å)		0.009
Bond angles (°)		1.459

Values in parentheses are for the highest-resolution shell.
[a] Diffraction data from a **single crystal** were used to obtain the structure.
[b] $I/\sigma I = 2$ at 2.70 Å resolution.

Table S4. DNA primers used for cloning and synthesis of DNA templates.

Primer name	Nucleotide sequence (5′ to 3′)
AflII-del-F	AACGAGAAAAATATAAAACACAGTCAAAAC
AflII-del-R	CATTATAACCCTCTTTAATTTGGTTATAATG
ErmC-Flag-C-F	GACTACAAAGACGATGACGACAAGTAAGAATTCTCTAGCCC
ErmC-GGGS-C-R	GGATCCGCCACCCTTATTAAATAATTTATAGCTATTGAAAAGAG
1ATG>ACG-R	CGTTATAACCCTCTTTAATTTGGTTATAATGAA
23ATG-exch-F	ACAAATATAAGATTAAATGAACATGATAATATC
23ATG>CTC-R	GAGTATTTTATCTATATTATGTTTTGAAGTAATAAAGT
T7ermCLC-fwn	CTGTACAT**TAATACGACTCACTATAGGG**AATTGTGAGCGGATAACAATTGC
T7ermC-fwn	CTGTACAT**TAATACGACTCACTATAGGG**AATTAAAGAGGGTTATAATGAACGAGAAA
T7ermCLC-rvn	CTCCGCTATCGCTACGTGAC
ErmBL-fwd	AC**TAATACGACTCACTATAGGG**CTTAAGTATAAGGAGGAAAAAATATGTTGGTATTCCAAATGCGTAATGTAGATAAAACATCTAC
ErmBL-rev	GGTTATAATGAATTTTGCTTATTAACGATAGAATTCTATCACTTTTTTTATTATTATTATTTCAAAATAGTAGATGTTTTATCTACAT
ErmCL-fwd	AC**TAATACGACTCACTATAGGG**AGTTTTATAAGGAGGAAAAAATATGGGCATTTTTAGTATTTTTGTAATCAGCACAGTTCATTATCAA
ErmCL-rev	GGTTATAATGAATTTTGCTTATTAACGATAGAATTCTATCACTTTTTTTATTATTATTATTTTTTGTTTGGTTGATAATGAACTGTGCT
ErmDL-fwd	AC**TAATACGACTCACTATAGGG**AGTTTTATAAGGAGGAAAAAATATGACACACTCAATGAGACTTAAGTTCCCAACTTTGAAC
ErmDL-rev	GGTTATAATGAATTTTGCTTATTAACGATAGAATTCTATCACTTTTTTTATTATTATTACTGGTTCAAAGTTGGGAAC
NV1	GGTTATAATGAATTTTGCTTATTAAC
RT	AAAACGCGTGTTAAATCCAT

The sequence of the T7 promoter is marked in bold; the sequence of the NV1 primer used for reverse transcription is underlined.

Table S5. Sequences of DNA templates used in toeprinting assay.

DNA template	Nucleotide sequence (5′ to 3′)
ermBL	AC**TAATACGACTCACTATAGGG**CTTAAGTATAAGGAGGAAAAAATATGTTGGTATTCCAAATGCGTAATGTAGATAAAACATCTACTATTTTGAAATAATAATAATAAAAAAAGTGATAGAATTCTATC**GTTAATAAGCAAAATTCATTATAACC**
ermCL	AC**TAATACGACTCACTATAGGG**AGTTTTATAAGGAGGAAAAAATATGGGCATTTTTAGTATTTTTGTAATCAGCACAGTTCATTATCAACCAAACAAAAAATAATAATAATAAAAAAAGTGATAGAATTCTATC**GTTAATAAGCAAAATTCATTATAACC**
ermDL	AC**TAATACGACTCACTATAGGG**AGTTTTATAAGGAGGAAAAAATATGACACACTCAATGAGACTTAAGTTCCCAACTTTGAACCAGTAATAATAATAAAAAAAGTGATAGAATTCTATC**GTTAATAAGCAAAATTCATTATAACC**

The sequence of the T7 promoter and the sequence complementary to the NV1 primer are marked in bold, respectively. Coding sequences are underlined.

Table S6. Parameters of the Toe-seq data sets.

Raw data sets, **before** normalization to untreated controls	
Total number of unique mRNAs in all 6 data sets	58,413
Total number of mRNAs in the AZI-1 data set (AZI replicate 1)	56,512
Total number of mRNAs in the AZI-2 data set (AZI replicate 2)	56,654
Total number of mRNAs in the AZI-BB2-1 data set (AZI-BB2 replicate 1)	56,657
Total number of mRNAs in the AZI-BB2-2 data set (AZI-BB2 replicate 2)	56,611
Total number of mRNAs in the Control-1 data set (Control replicate 1)	55,835
Total number of mRNAs in the Control-2 data set (Control replicate 2)	56,089
Total number of mapped reads in the AZI-1 data set (AZI replicate 1)	9,316,067
Total number of mapped reads in the AZI-2 data set (AZI replicate 2)	11,236,276
Total number of mapped reads in the AZI-BB2-1 data set (AZI-BB2 replicate 1)	10,171,724
Total number of mapped reads in the AZI-BB2-2 data set (AZI-BB2 replicate 2)	10,326,046
Total number of mapped reads in the Control-1 data set (Control replicate 1)	6,148,055
Total number of mapped reads in the Control-2 data set (Control replicate 2)	5,923,640
Data sets **after** normalization to untreated controls, **before** filtering the data	
Total number of unique mRNAs in 4 data sets	56,636
Total number of unique mRNAs in 2 data sets (AZI replicates 1 and 2)	56,286
Total number of unique mRNAs in 2 data sets (AZI-BB2 replicates 1 and 2)	56,280
Total number of mRNAs in the AZI-1 data set (AZI replicate 1)	55,225

Total number of mRNAs in the AZI-2 data set (AZI replicate 2)	55,493
Total number of mRNAs in the AZI-BB2-1 data set (AZI-BB2 replicate 1)	55,283
Total number of mRNAs in the AZI-BB2-2 data set (AZI-BB2 replicate 2)	55,476
Number of shared mRNAs between two data sets (AZI replicates 1 and 2)	54,432
Number of shared mRNAs between two data sets (AZI-BB2 replicates 1 and 2)	54,479
Number of mRNAs with coincident stalling sites in two data sets (AZI replicates 1 and 2)	11,097
Number of mRNAs with coincident stalling sites in two data sets (AZI-BB2 replicates 1 and 2)	14,357
Data sets **after** normalization to untreated controls and **after** filtering the data	
Total number of unique mRNAs in 4 data sets	30,174
Total number of unique mRNAs in 2 data sets (AZI replicates 1 and 2)	23,283
Total number of unique mRNAs in 2 data sets (AZI-BB2 replicates 1 and 2)	23,018
Total number of mRNAs in the AZI-1 data set (AZI replicate 1)	14,312
Total number of mRNAs in the AZI-2 data set (AZI replicate 2)	15,280
Total number of mRNAs in the AZI-BB2-1 data set (AZI-BB2 replicate 1)	14,135
Total number of mRNAs in the AZI-BB2-2 data set (AZI-BB2 replicate 2)	15,344
Number of shared mRNAs between two data sets (AZI replicates 1 and 2)	6,309
Number of shared mRNAs between two data sets (AZI-BB2 replicates 1 and 2)	6,461
Number of mRNAs with coincident stalling sites between two data sets (AZI	1,911

replicates 1 and 2)	
Number of mRNAs with coincident stalling sites between two data sets (AZI-BB2 replicates 1 and 2)	2,619

SUPPLEMENTARY REFERENCES

1. Osterman IA, Komarova ES, Shiryaev DI, Korniltsev IA, Khven IM, Lukyanov DA, Tashlitsky VN, Serebryakova MV, Efremenkova OV, Ivanenkov YA, Bogdanov AA, Sergiev PV, Dontsova OA. 2016. Sorting Out Antibiotics' Mechanisms of Action: a Double Fluorescent Protein Reporter for High-Throughput Screening of Ribosome and DNA Biosynthesis Inhibitors. *Antimicrob Agents Chemother* 60:7481–9.
